# Microplastic Identification Methods for Microfluidic Applications: Towards Rapid Detection in Aquatic Environments

**DOI:** 10.3390/polym18151847

**Published:** 2026-07-28

**Authors:** Camila Maria Penso, Maria C. Paiva, José Viana-Gomes, Luís M. Gonçalves

**Affiliations:** 1 CMEMS, Department of Industrial Electronics, University of Minho, 4800-058 Guimaraes, Portugal; 2 Department of Polymer Engineering, Institute for Polymers and Composites, University of Minho, 4800-058 Guimaraes, Portugal; 3 Department of Physics, Centre for Physics, University of Minho, 4710-057 Braga, Portugal; zgomes@fisica.uminho.pt; 4 LABBELS–Associate Laboratory, University of Minho, 4710-057 Braga, Portugal; lgoncalves@dei.uminho.pt

**Keywords:** marine environment, microplastics, polymer characterization, FTIR spectroscopy, Raman spectroscopy, machine learning, Lab-on-a-Chip, environmental monitoring

## Abstract

The escalating accumulation of microplastics (MPs) in marine ecosystems presents a critical environmental crisis. However, current monitoring efforts rely heavily on labor-intensive, contamination-prone, and time-consuming laboratory analyses. While these conventional off-chip methods provide high accuracy, they inherently lack the throughput and autonomy required for continuous, real-time oceanic surveillance. To bridge this technological gap, microfluidic technologies (Lab-on-a-Chip) provide a viable route towards miniaturized, reagent-free in situ detection with reduced sample volumes and continuous operation capability. This review examines the transition from benchtop to field-deployable platforms and organizes the available microfluidic approaches for MP analysis into a structured overview. We examine on-chip sample manipulation and complementary separation techniques, such as acoustophoresis, dielectrophoresis, and optical tweezers, which are essential for isolating target particles from complex environmental matrices and overcoming intrinsic microfluidic challenges. Following sample preparation, we provide a comprehensive evaluation of state-of-the-art optical and spectroscopic identification methods optimized for continuous flow detection. Finally, we address current analytical limitations and discuss how the integration of machine learning with dynamic spectral libraries could enable autonomous, field-deployed monitoring networks for long-term MP surveillance.

## 1. Introduction

Microplastics (MPs) have been detected in virtually every aquatic compartment sampled to date, from open ocean gyres to deep-sea sediments, making their accumulation one of the most pervasive and poorly quantified pollution problems in current marine research. This introduction outlines the origins and environmental relevance of MPs, examines the diversity and distribution of polymers across marine ecosystems, and critically contrasts the limitations of conventional laboratory analysis with the urgent need for in situ monitoring technologies.

### 1.1. Context and Relevance of MP Pollution

**Figure 1:**
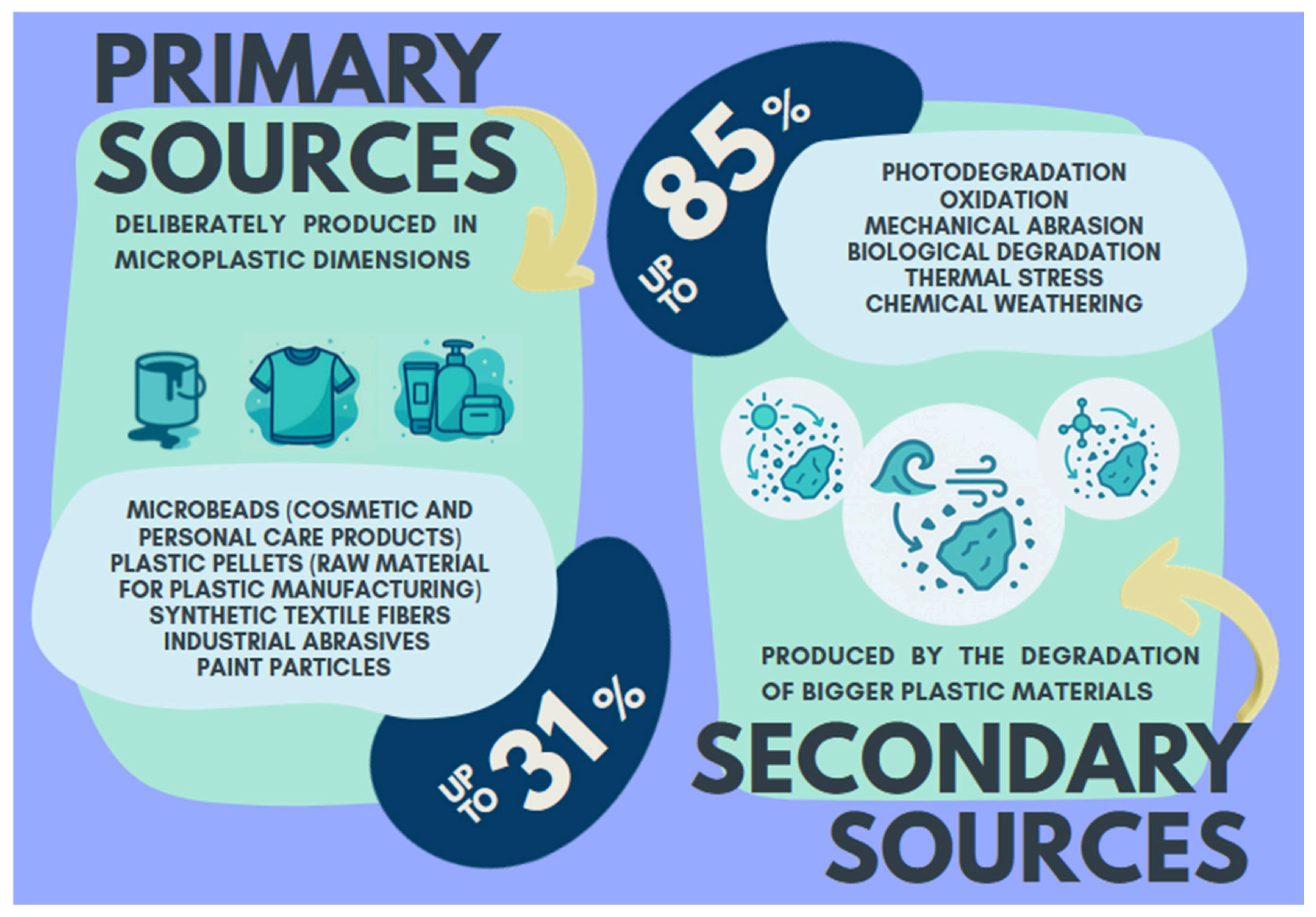
Primary vs. secondary MPs.

Large-scale plastic production began in the 1950s and has grown exponentially ever since [1]. Despite this relatively short timespan, the ocean is currently estimated to harbor more than 250,000 tonnes of plastic floating at the surface alone, comprising over five trillion individual pieces [2], ranging from macroplastics (>2.5 cm) to MPs (1 μm–5 mm) [3]. MPs arise from either primary (up to 31%) or secondary sources (up to 85%) [4] (see Figure 1). They are either intentionally manufactured at the microscale or result from the environmental fragmentation of larger plastic items [5]. Secondary MPs are more difficult to mitigate because they emerge from slow and unpredictable breakdown processes. Laboratory degradation rates consistently overestimate field conditions, where biofouling, low temperatures, and sediment burial collectively slow fragmentation and sustain particle loads over decadal timescales [6].

MPs are now found in almost every part of marine and land ecosystems, ultimately finding their way into the human food chain and becoming an omnipresent source of contamination [7]. Detection has occurred in obvious vectors such as seafood (fish [8], crustaceans [9], bivalve mollusks [10] and table salt [11]) but also in less expected items, including bottled water and beverages [12], beer [13], milk and dairy products [14], vegetables irrigated with contaminated water or grown in plastic-mulched soils [15], and even meat and poultry [16].

Beyond ingestion, human exposure occurs through multiple pathways, namely, inhalation of airborne fibers and fragments [17], dermal contact [18], and transplacental transfer, with MPs already identified in human placenta, meconium and umbilical cord blood [19,20]. Once inside the body, MPs can translocate across biological barriers and accumulate in organs, where they have been associated with a range of toxic effects, including oxidative stress, endocrine disruption, and developmental impairment [21]. A particularly concerning threat arises from the microbial communities that colonize MPs. These biofilms host a diverse community of pathogenic and antibiotic-resistant bacteria, along with potentially harmful fungi [22,23,24]. MPs facilitate the long-range oceanic dispersal of these pathogens and resistance genes in the marine environment and in human-associated microbiomes [25]. The combination of chemical toxicity and microbial dispersal makes MPs a particularly complex and multifaceted threat.

### 1.2. Polymer Diversity and Distribution in Marine Ecosystems

**Figure 2:**
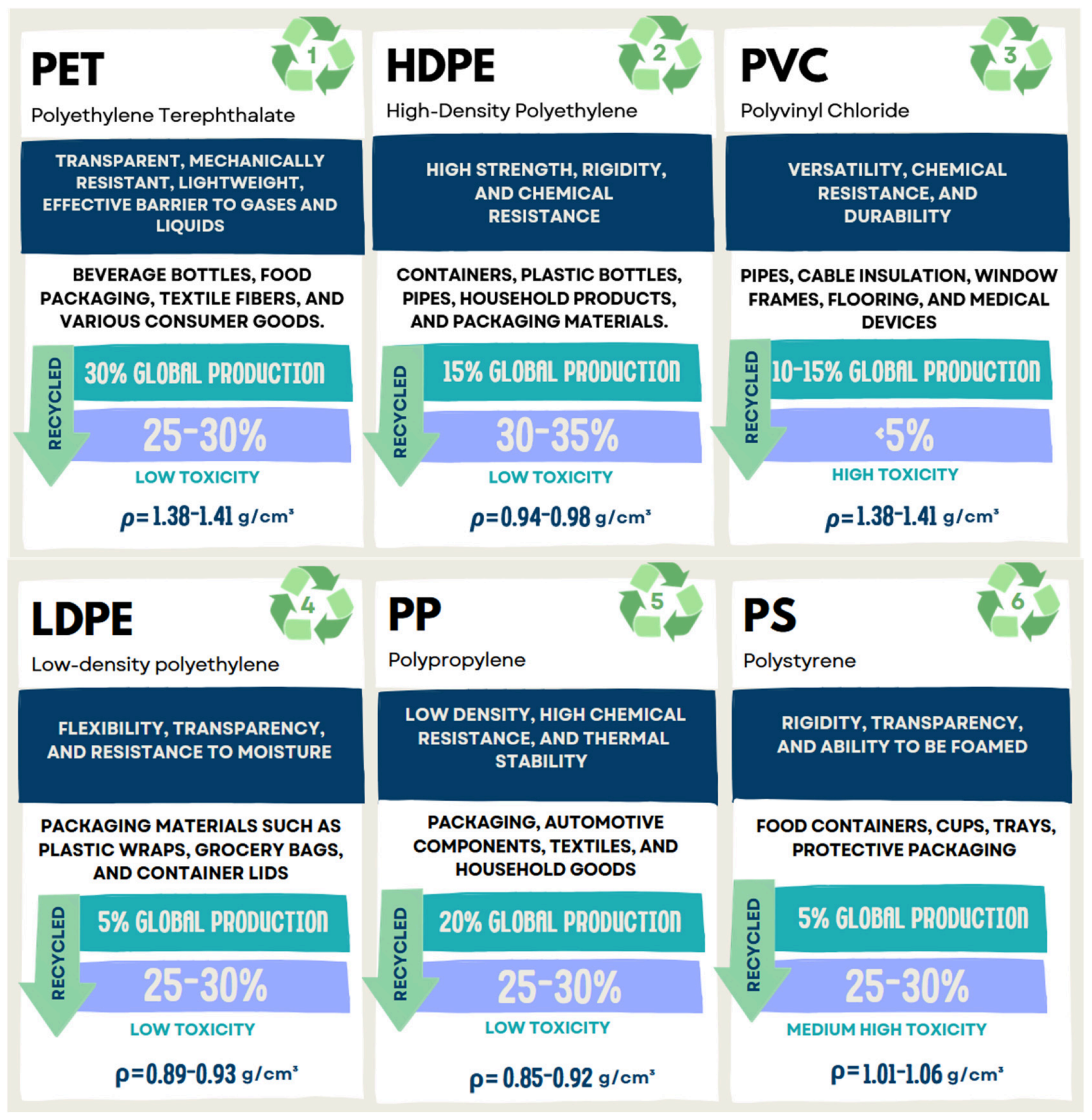
Characteristics of the main types of polymers; density from [27].

The oceanic distribution of MPs, whether they predominantly occur at the sea surface, suspended in the water column, or settled on the seafloor, is still the subject of intense research [26]. Polymer density is the primary determinant of vertical position in the water column. As shown in Figure 2, commonly detected polymers display a wide range of densities. This polymer diversity directly translates into distinct buoyancy behaviors in seawater. However, long-term environmental weathering, biofouling, and fragmentation progressively modify the density of particles, often causing initially floating polymers to sink and denser ones to become incorporated into sinking aggregates. Consequently, the same polymer type can be found across multiple marine environments [27,28].

The chemical makeup of these polymers intensifies their physical persistence with direct toxicological risks. While the base polymer matrix is relatively inert, degradation releases co-formulated additives (e.g., plasticizers, stabilizers, pigments) that carry significant toxicological loads [29,30]. For instance, PVC can leach phthalates and organotin stabilizers, PS can release brominated flame retardants and phthalates, and PET is associated with antimony trioxide, a suspected carcinogen employed as a polymerization catalyst [31,32,33]. Compounding this issue, the extremely low global recycling rates of most marine-relevant polymers contribute to massive annual inputs of virgin plastic and mismanaged waste into the oceans [34].

Although MPs undergo diverse physical and chemical transformations in the environment, a clear morphological hierarchy emerges from global studies: fibers are consistently the most abundant shape, followed by fragments, films, pellets/beads, and, to a lesser extent, foams [27,35,36]. In parallel, particle size represents another critical dimension in MP research. Although most studies define MPs as particles ranging from 1 μm to 5 mm, a pronounced detection bias favors larger fractions, as over 80% of field studies employ nets with mesh sizes ≥ 300 μm, which systematically exclude the smaller size fraction from datasets [37]. When finer meshes (<100 μm) or alternative techniques are used, detected concentrations increase substantially, confirming that small MPs are present but routinely missed rather than genuinely scarce. Furthermore, smaller particles tend to exhibit more regular morphologies than larger fragments, which often display highly irregular, angular edges resulting from prolonged mechanical and photo-oxidative degradation [38].

Along the Catalan coast, blue and transparent particles are consistently the dominant colors, reflecting the prevalence of weathered polyolefin fragments and synthetic textile fibers. Fragments dominate MPs larger than 0.5 mm (58%), while fibers account for 70% of the smaller fraction (<0.5 mm), composed predominantly of polyester (37%) and polyamide (24%) [39]. In contrast, surveys in the western Pacific Ocean reveal a markedly different profile: white (34%), transparent (31%), and green (25%) are the most common colors [40], with fibers/filaments predominant (57% of total MP) and abundances ranging from 0.03 to 2.36 particles·m^−3^ depending on proximity to subtropical gyres and major riverine outflows [40,41].

In European surface water samples, the most abundant plastic polymers are PP, PE, PS, PET, and PE fibers, with reported concentrations typically ranging from 0.1 to 600 particles·m^−3^, reflecting improved wastewater treatment efficiency and monitoring sensitivity [42]. In the western Pacific Ocean, PP and PE are consistently the most abundant polymer types, with PP alone accounting for up to 39% of identified particles [40]. A time-series survey spanning 2008–2018 in the Eastern Tropical Pacific further documented a significant and sustained increase in MP abundance over this period, with PE and PP again identified as the dominant polymers [43].

### 1.3. Laboratory vs. In Situ Analysis

The vast majority of MP prevalence studies rely on laboratory analysis of collected samples. Nevertheless, upon transferring the collected sample from the site, a potential for cross-contamination emerges, involving interactions between the sample and laboratory materials, reagents, and chemical substances.

A potential source of overestimation in MP analysis arises from contamination within the laboratory reagents themselves. Kutralam-Muniasamy et al. [44] reported the presence of MP particles, predominantly fibers and uncolored particles composed of PE, PP, and polyester, in several commonly used reagents, meaning that MPs introduced through the reagents may be inadvertently counted as part of the sample, leading to inflated abundance estimates. On the other hand, the filters employed to extract a sample from liquid media also introduce the possibility of sample contamination [45]. Prata et al. [45] compiled a set of recommended procedures for laboratory work when conducting MP analysis. This includes specific emphasis on the protocol involving filters to mitigate the previously mentioned problem. In the scenario of in situ analysis, direct interaction with the marine environment occurs, minimizing disruptions from external variables. This reduces contamination risk and cuts costs associated with sample collection, laboratory processing, and specialized personnel.

During the analysis of samples within the marine environment, the accessibility of sampling sites is not consistently straightforward. Sometimes, due to geographical and oceanic conditions, it requires costly vessels, and access is not always feasible. Consequently, autonomous sensors designed for in situ monitoring prove to be optimal for prolonged data collection in demanding environments.

Satellite-linked sensors allow remote operators to monitor instrument status and adjust sampling parameters without physical access to the deployment site. Autonomous underwater vehicles equipped with such sensors can map the full 3D water column for MPs.

## 2. Microfluidics

Lab-on-a-Chip (LOC) microfluidic systems miniaturize and integrate sample manipulation, separation, and detection steps that conventional workflows distribute across multiple instruments and operator-intensive procedures. The transition from controlled laboratory environments to continuous in situ oceanic surveillance, however, introduces a unique set of operational hurdles. The specific challenges this transition introduces (material compatibility, biofouling, spectral interference) are examined in the following subsections.

### 2.1. Challenges in Microfluidic Applications

Sample preparation is a near-universal step in MP identification workflows, and its design has direct consequences for detection outcomes [46]. The primary objective of this step is to eliminate organic matter and contaminants from the sample. However, this process can occasionally damage the particles, resulting in changes to their shape and integrity [47]. The methodology used for this pretreatment has a direct impact on the detection results [48].

The different microfabrication techniques used for the fabrication of microfluidic devices are not described here but can be reviewed in [49,50,51]. A recurring operational challenge in microfluidic systems is fouling [52,53]. This occurs when particles in the sample accumulate on the walls of the microfluidic channel. Such fouling depends on the material composition of the channels and the nature of the sample, and it can have a negative impact on the accuracy and reproducibility of the readings. Channel geometry can further exacerbate this problem [54]. Beyond fouling, several operational challenges must also be addressed for microfluidic MP analysis. Given the vast diversity and abundance of organic and inorganic particles in environmental samples, the separation of MPs from other suspended matter remains a crucial yet complex task. Moreover, because the concentration of MPs in oceanic environments is typically very low, it is necessary to incorporate pre-concentration mechanisms to improve detection efficiency and analytical sensitivity. Once within the microfluidic channel, each particle must be isolated and immobilized for individual analysis, ensuring that optical or spectroscopic readings are not influenced by nearby debris or fluid turbulence. Following characterization, efficient particle removal or disposal is essential to prevent cross-contamination between consecutive analyses.

Long-term operational stability presents an equally serious constraint, particularly for deployments in uncontrolled field environments. Clogging, caused by particle aggregation, adhesion, or channel narrowing, can significantly alter flow dynamics, disrupt pressure balance, and reduce device lifespan. In parallel, biofouling, which results from the adsorption of biomolecules or microbial growth on channel surfaces, can further degrade sensor performance by blocking light paths, altering surface properties, and inducing unpredictable signal drift [55].

Mitigating these combined effects requires coordinated design decisions spanning channel materials, surface chemistry, and automated cleaning protocols, none of which can be treated in isolation for deployments intended to run continuously over weeks or months.

In microfluidic systems for MP analysis, channel material selection directly determines the risk of secondary contamination and compatibility with optical detection techniques.

Thermoplastic polymers frequently employed in rapid prototyping, such as poly(methyl methacrylate) (PMMA), PS, and polycarbonate (PC), exhibit strong intrinsic autofluorescence and Raman background signals that can overlap with common MP Raman bands (1000–1600 cm^−1^) and fluorescent labels, thereby reducing signal-to-noise (SNR) ratios in μ-Raman and fluorescence microscopy. Polydimethylsiloxane (PDMS), the silicone elastomer most widely used in microfluidic fabrication, presents the same limitation despite belonging to a chemically distinct material class [56,57]. PMMA and PS show higher autofluorescence levels compared to alternatives like cyclic olefin copolymer under common excitation wavelengths (400–800 nm) [58]. Borosilicate glass and fused silica remain widely regarded as gold standards owing to their excellent optical transmission from UV to near-IR (190–2500 nm), minimal background fluorescence, and chemical inertness, which minimize interference in MP detection [59,60,61].

### 2.2. Water Absorption Spectra

**Figure 3:**
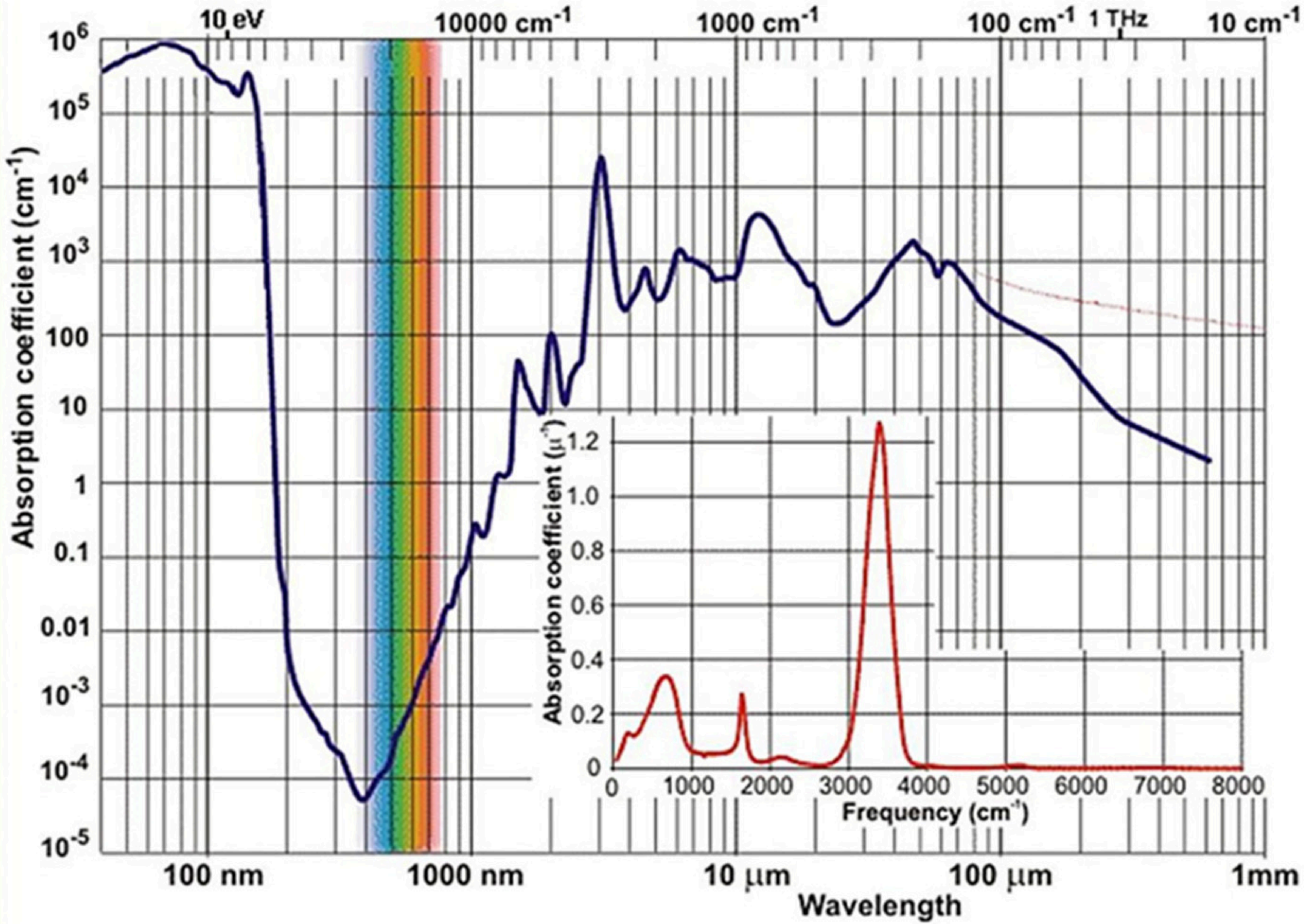
The spectrum of the absorption coefficient of liquid water [62].

Detecting suspended particles directly within an aqueous microfluidic stream is termed in-line analysis. In-line analysis poses significant challenges. The molecular bonds of water originate absorption peaks in the same spectral region as those of most MP materials; thus, the absorption spectrum of water may overlap with that of the sample, leading to potential misinterpretations. In the water absorption spectrum, a major peak near 3400 cm^−1^ (2900 nm) is associated with O-H stretching, as can be observed in Figure 3 [62]. Considering the small path of the beam in water (typically below 1 mm), wavelengths between 200 nm and 2000 nm can be used, but larger attenuation is expected above 2000 nm.

### 2.3. Interfering Particles in Environmental Water Samples

Accurate identification of MPs in environmental samples is frequently compromised by the presence of nonplastic particulate matter. Nonplastic particles can account for 15–30% of all suspected particles detected in studies, underscoring the need for rigorous chemical validation to avoid false positives [63].

Carbon black strongly interacts with suspended particles through aggregation and adsorption onto sinking particles, thereby altering sedimentation dynamics [64]. Carbon black absorbs light in the visible and near-infrared ranges, which causes signal overlap in Raman and Fourier transform infrared (FTIR) spectroscopy, leading to misidentification of MPs [65]. Natural fibers and anthropogenic cellulose particles commonly co-occur with MPs in marine and estuarine ecosystems, complicating selective detection [66]. In the Galápagos Islands, for instance, cellulose particles were detected at concentrations comparable to those of synthetic MPs, including within the digestive tracts of the endangered Galápagos penguin [66]. Aggregation of MPs with mineral particles is widely documented in both freshwater and marine systems. These interactions increase the effective density of aggregates and accelerate the sedimentation of MPs, particularly in the <100 μm size fraction [67,68]. Mineral and natural organic debris frequently adhere to polymer particles or morphologically mimic them, thus requiring rigorous chemical digestion and spectroscopic validation to minimize false positives [69]. Table 1 summarizes the major spectral interferences by nonplastic particles documented in the literature.

### 2.4. Particle Counting, Sorting and Detection Techniques

The subsequent subsections describe techniques that primarily serve to count, size, and physically separate MPs from other suspended matter, rather than chemically identify polymer type. Several of these methods are nonetheless directly compatible with downstream spectroscopic identification when used as upstream sample manipulation steps and are therefore essential components of integrated microfluidic platforms. A comprehensive summary of recent studies applying these techniques is provided in Table 2.

**Table 1:** Summary of major spectral interferences in MP analysis.

Interferent	Typical Size Range	Key Spectral Interference		Ref.
Carbon Black (soot, charcoal)	Primary particles: 20–100 nm Aggregates: 0.5–10 μm	Raman	The strong D band (1350 cm^−1^) and G band (1580 cm^−1^) tend to dominate the fingerprint region, masking polymer-specific signals.	[63,70,71]
		FTIR	Produces a broad, featureless rising baseline across the entire mid-IR range.	
Natural Fibers (cellulose, cotton, wood, paper)	Length: 100 μm—several mm Diameter: 5–50 μm	Raman	Cellulose produces a relatively weak Raman signal, but its peaks at 1095 and 1120 cm^−1^ can overlap with those of PET, potentially leading to misidentification in mixed samples.	[66,71,72,73]
		FTIR	A broad O–H stretching band (3300–3400 cm^−1^) and strong glycosidic C–O–C absorptions (1000–1200 cm^−1^) are characteristic of cellulose and can partially overlap with synthetic polymer bands.	
Minerals (quartz, clays, feldspar, carbonates)	<2 μm (clays) >1 mm (sand)	Raman	Quartz produces a sharp, intense band at 464 cm^−1^ that can dominate spectra of mineral-rich samples. Clays show broad Al–OH stretching bands in the 3600–3700 cm^−1^ region.	[71]
		FTIR	The Si–O stretching mode (1000–1100 cm^−1^) is very strong and overlaps with several common polymer bands. Additional bending modes appear in the 400–550 cm^−1^ region.	
Proteins/Amorphous Organics	0.5–100 μm	Raman	Organic matter frequently induces strong fluorescence that can overwhelm the Raman signal entirely. When fluorescence is manageable, the amide I band (1655 cm^−1^) may interfere with signals from some polymers.	[72,74,75]
		FTIR	Amide I (1650 cm^−1^) and amide II (1550 cm^−1^) absorption bands are present in proteinaceous material and can complicate identification of plastics with overlapping carbonyl or C–N features.	
Diatoms/Biogenic Silica	5–200 μm	Raman	Diatom frustules display silica-related Raman bands that may overlap with some polymer signals. Chlorophyll a, often present in live or recently deceased diatoms, contributes strong autofluorescence and characteristic peaks that can mask plastic signatures.	[72]

#### 2.4.1. Electrical Impedance Spectroscopy

Electrical impedance spectroscopy (EIS) is a technique that measures the electrical impedance of a sample at various frequencies, providing information on its resistance, capacitance, and inductance. The impedance values depend not only on the size and morphology of the particle but also on the electrode geometry and microfluidic channel design [76,77]. Typically, this technique involves coplanar or parallel electrodes within a microfluidic channel through which the sample flows. One pair of electrodes is driven by a sinusoidal wave whose frequency varies over time. As a particle passes through the electrodes, it alters the measured impedance, with the impedance change being proportional to the particle volume at low frequencies and reflecting both the material properties and volume of the sample at high frequencies.

This methodology is used to measure MPs in aqueous matrices [78,79]. The use of impedance spectroscopy offers distinct advantages over other methods, as the equipment is simpler, less expensive, and less prone to misidentification with organic matter. However, one of the drawbacks of impedance spectroscopy is its tendency to identify air bubbles as plastic, owing to similarities in dielectric constants. To optimize this process, it is necessary to use methods that centralize particles within the channel and prevent multiple particles from passing simultaneously.

In a study by Colson and Michel [80], MP recovery rates higher than 90% were achieved for particle sizes ranging from 300 to 1000 μm. However, for particle sizes between 212 and 250 μm, recovery rates dropped by half. Nonetheless, the authors suggested that recovery rates for particles smaller than 300 μm could be improved by adding an additional electrode and reducing the spacing between electrodes. Although this method enables the differentiation of plastics from other components, such as organic matter, its sensitivity in distinguishing different polymers is not as robust as other methods, such as Raman or FTIR spectroscopy. As such, it may be an excellent complement to a detection system [81].

#### 2.4.2. Resistive Pulse Sensor

A resistive pulse sensor (RPS), also known as a Coulter counter, is a system that enables counting and measuring particle sizes within a microfluidic channel. When a particle passes through the channel, it induces a change in electrical resistance between two electrodes [82]. This change can be measured, and the read signal is proportional to the volume of the identified particle. M. Pollard et al. [83] successfully detected PS particles measuring 2–30 μm in size across a range of salt concentrations, with a minimum detectable concentration of 14 particles·mL^−1^ at a pressure of 100 mbar.

#### 2.4.3. Acoustophoresis

When utilizing acoustic pressure to manipulate the motion of particles, the process is termed acoustophoresis. This can be achieved through the utilization of three distinct types of waves, namely, bulk acoustic waves (BAWs), surface acoustic standing waves (SASWs), and acoustic traveling waves (ATWs). The effective application of the first two methods requires a density disparity between the particles and the surrounding medium. BAW is the most widely used variant, operating without labels or electrodes: a piezoelectric transducer (PZT) drives a vibrational wave through the microchannel wall into the fluid, generating a standing acoustic pressure field that displaces particles towards nodal positions. It can be used to separate particles according to the acoustic contrast factor (ACF), which depends on both density and compressibility relative to the surrounding medium, to merge fluorescent dye droplets with other relevant droplets or to center particles in a microchannel [84].

Akiyama et al. [84] demonstrated acoustophoretic focusing of PS spheres (15 μm diameter) and fibrous PA and PET MPs (200 μm length, <10 μm diameter) in a trifurcated glass microchannel, achieving near-complete collection of particles in the central outlet. Nonetheless, they acknowledged that this method could present challenges when applied to a heterogeneous mixture of microparticles or samples containing high levels of organic matter.

#### 2.4.4. Dielectrophoresis

Dielectrophoresis (DEP) involves separating polarizable particles suspended in a fluid using a non-uniform electric field. This method is applied in various studies on microfluidics [85,86]. It enables sample analysis without the need for pretreatment and, unlike alternative techniques, it does not produce misleading results in the presence of biofilm-laden particles. Additionally, this method can separate MPs from naturally occurring particles and has the potential to sort polymers by type. DEP alone does not provide chemical identification of polymer type and must therefore be coupled with a downstream spectroscopic method.

#### 2.4.5. Optical Tweezers

**Figure 4:**
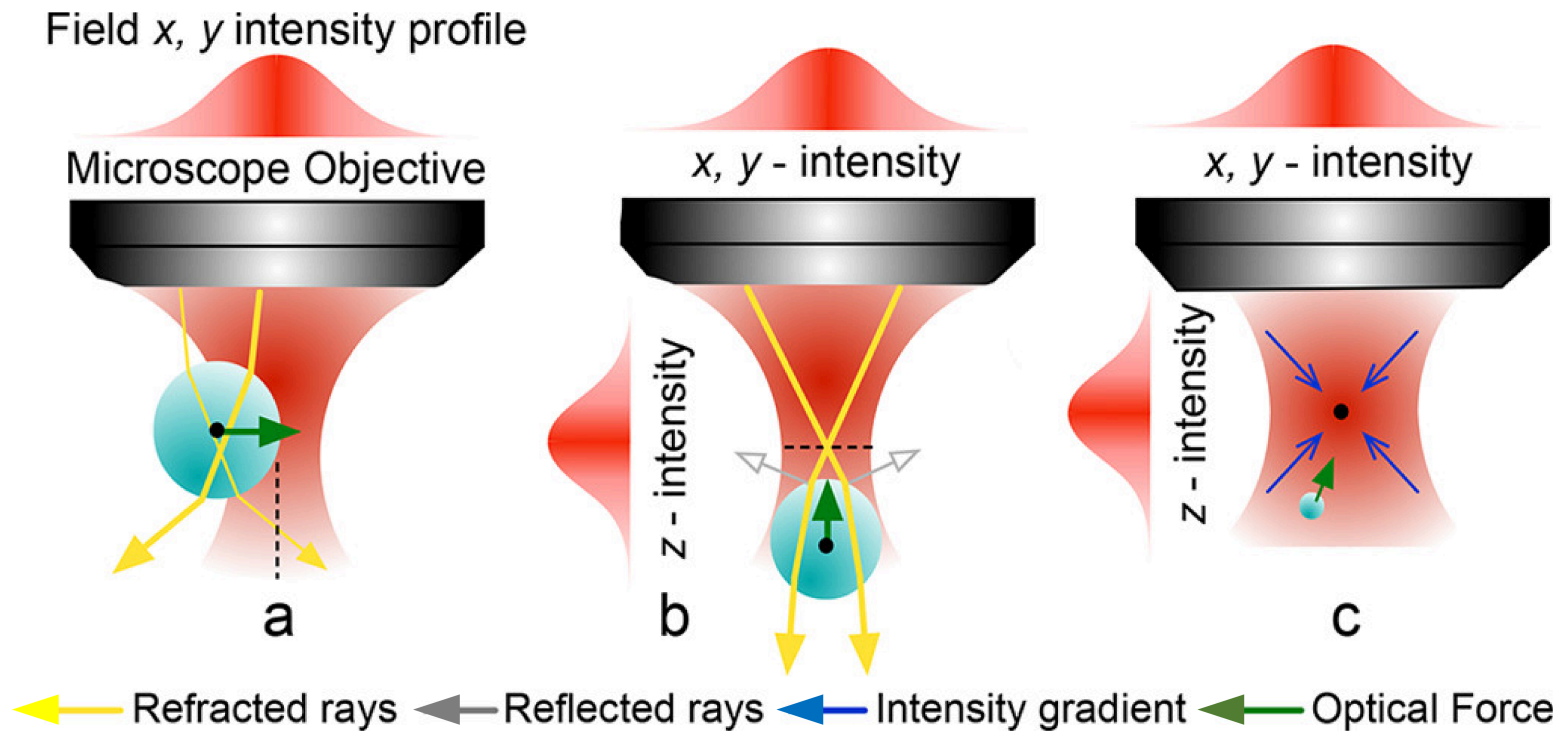
Diagram illustrating the principle of OT into the ray optics regime (**a**,**b**), for MPs down to several microns in size, and in the Rayleigh regime (**c**), valid for NPs [89].

Optical tweezers are a powerful tool for manipulating microscopic particles using a highly focused laser beam capable of exerting gradient forces on transparent materials. The focused beam generates an optical potential well that traps a particle, enabling its controlled manipulation and subsequent analysis (Figure 4). As a fully contact-free technique, it eliminates the risk of sample contamination or physical alteration. This approach allows the isolation of individual transparent MPs even within complex matrices such as environmental suspensions or biological samples.

Several identification techniques require significant processing time for the signal readout, making particle immobilization within the channel desirable. Microfluidic traps are frequently utilized to obtain more reliable spectra in such cases. The types of traps used to immobilize particles in the channel, including optical, mechanical, electric potential difference, electromagnetic, acoustic, or magnetic traps, are described in [87]. The choice of trap depends on factors such as fluid velocity, channel geometry, particle size, and sample type being analyzed.

Recently, optical traps have been extensively used, especially in conjunction with Raman technology [88]. Recent investigations detail the utilization of optical tweezers [89], specifically aimed at identifying MPs and NPs in the sub-20 μm fraction, with identification demonstrated down to 50 nm aggregates in distilled water and sub-micron particles in seawater. Xu et al. [90] successfully trapped and chemically identified microplastics directly in real freshwater samples without any chemical pretreatment. In seawater matrices, Ripken et al. [91] achieved reliable identification of both free-floating particles and those embedded in organic matter, noting additional Raman features in smaller, weathered fragments. Kazemi et al. [92] investigated trapping stability across different polymers, finding that PP particles exhibited the highest stability, whereas PET particles larger than 10 μm were significantly more difficult to maintain in the optical trap, particularly those that were darker or exhibited greater light absorption. These approaches enable the stabilization of particles to facilitate Raman readings, which can occasionally take several minutes to complete. Extended reading times typically correlate with heightened sensitivity and selectivity.

#### 2.4.6. Field-Flow Fractionation

Field-flow fractionation (FFF) is a particle separation technique that employs a field (gravity, centrifugation, or crossflow) applied perpendicular to the flow of water through a narrow channel. This field drives particles toward the channel wall, resulting in a vertical distribution according to particle size: larger particles remain closer to the wall, where the fluid moves more slowly, while smaller particles occupy higher positions, where the flow is faster. Therefore, particles travel at different velocities and exit the channel at distinct times, achieving natural size-based separation.

**Table 2:** Overview of complementary techniques for MP identification.

Technique	Particle Size Range	Target Plastics	Matrix	Performance Metrics	Ref.
EIS	20–30 μm (PVC) 194–542 μm (PE)	PVC, PE	Water solutions	Both polymer types were reliably distinguished and quantified based on their surface charge. PVC produced stronger impedance signals than PE. Concentration estimation error remained below 3% across the full frequency range for PVC. PE detection was possible but with lower sensitivity.	[78]
	500–4000 μm	PET	Water solutions	EIS signals were sensitive to PET particle size, enabling discrimination between four size fractions in complex matrices containing organic and inorganic matter. Detection was dependent on particle proximity to the electrodes, limiting sensitivity for smaller particles. ML postprocessing was required to achieve classification, with best accuracy of 89.2% in complex water matrices.	[79]
RPS	2–30 μm	NR	Water solution	The 3D-printed sensor could be tuned in real time by adjusting channel geometry, covering a size range of 2–30 μm. MPs produced a distinctive conductive pulse, clearly distinguishable from the resistive signal of algae and smooth calibration beads, allowing rapid confirmation of plastic presence. The minimum detectable concentration was 14 particles·mL^−1^ at 100 mbar, with a throughput of 1 mL/min.	[83]
	200 nm	PS (calibration bead)	Water solution	Size and concentration characterization of 200 nm PS particles was achieved with less than 5% error. Multiple detection gates combined with hydrodynamic sheathless focusing reduced noise and improved SNR. Throughput exceeded 200,000 particles per second. The system was developed primarily for biological nanoparticles; PS beads served as calibration standards only.	[93]
Acoustophoresis	PS (15 μm) PET and PA (200 μm length < 10 μm diameter)	PS, PA, PET	Water solution	Collection efficiencies were high across all tested plastics: 99% for PS spheres, 99% for Nylon 6 (PA) fibers, and 95% for PET fibers. Theoretical modeling suggests the system could collect particles down to 4.3 μm (PS), and potentially 1 μm with further design refinements. However, rigid organic debris with positive acoustic contrast factors may co-collect with MPs, limiting selectivity in complex environmental samples.	[84]
Optical Tweezers (Paired with Raman)	1.1–25.4 μm	PS, PE, PVC, NY6 and PMMA	Surface freshwater samples	MPs were successfully trapped and chemically identified in real freshwater samples without chemical pretreatment. Of 514 particles, 136 (26.5%) were MPs, with PS as the dominant polymer (63.2%). The smallest particle identified was 1.1 μm PS.	[90]
	Sub-20 μm, up to 50 nm	PS, PP, PE, PET, PVC, PMMA, PA6	Distilled water and seawater	MPs and NPs were optically trapped and chemically identified in both distilled water and seawater. In seawater, particles of PE, PET, PVC and PP were unambiguously identified at the single-particle level, discriminated from mineral sediments and organic matter. Analysis on naturally aged PE and PP fragments confirmed the method’s applicability to environmental samples.	[89]
	1.4–47.8 μm	PE, PP, PVC, PA, PS	Seawater	Most MPs were embedded in organic matter (68.75%), with only 31.25% free-floating. No chemical sample preparation was required. Additional Raman peaks with decreasing particle size indicated structural modification from weathering or organic embedding.	[91]
	1–50 μm	PP, PET, HDPE	Water	Trapping stability varied considerably between polymers: PP was the most stable, while PET particles above 10 μm were three times less likely to remain trapped. HDPE showed intermediate behavior. Darker or more absorptive particles were generally harder to trap due to their optical properties.	[92]
FFF (AF4—Asymmetric Flow FFF)	100 nm (PS) 1–100 nm (PE)	PS, PE	Spiked fish fillet	PS nanoplastics were successfully separated and detected after enzymatic digestion. PE particles were detectable in aqueous solution but could not be detected in fish due to an elevated LS background from the matrix. Results demonstrated that methods optimized for one polymer type may not be directly applicable to others.	[94]
	200 nm–5 μm	PS, PMMA	Aqueous suspension	Minimum detectable concentrations ranged from 10 μg/L (PS 600 nm) to 200 mg/L (PS 200 nm), with a typical sensitivity around 1 mg/L (10^9^ L^−1^). Particles of 100 nm did not produce a detectable Raman signal. Optical tweezers were required to retain particles sufficiently long for spectrum acquisition. Both AF4 and CF3 were demonstrated.	[95]
	20–200 nm	PS	Aqueous standard solutions	AF4 separated four PS-NP sizes in 48 min using 0.2% SDS carrier. Recovery was 91–99% across all sizes. Trueness on size was 102–112%. Peak resolution between consecutive fractions was 1.5–2.7. Between-day repeatability was ca. 10% RSD. LOD by UV was 15–33 μg/mL (37.5–87.5 ng injected). MALS could not size particles with Rg below 10 nm.	[96]
	100–500 nm	PS	UHT skim milk	AF4 successfully separated PS nanoplastics from UHT skim milk components. Recovery for PS300 and PS500 in milk was >90%. Milk stability in the AF4 channel was confirmed over 48 h. PS100 was detectable but could not be fully separated from caseins. First proof of applicability of AF4-RM to a real food matrix.	[97]

NR—not reported.

## 3. Identification Methods

The separation and pre-concentration techniques described in Section 2 are prerequisites for reliable MP identification, but they do not yield chemical information. Polymer assignment relies on spectroscopic or optical methods that generate molecular fingerprints specific enough to discriminate between chemically similar materials under realistic environmental conditions. This section reviews the principal identification techniques reported in the literature for MP analysis, evaluating their sensitivity, spatial resolution, water compatibility, and suitability for integration into microfluidic platforms.

The absence of a standardized analytical protocol remains a persistent obstacle, and methodological heterogeneity across studies makes quantitative comparisons unreliable and impedes the construction of coherent global datasets. A recent coordinated effort to harmonize quality assurance procedures for MP sampling and measurement [3] represents a significant step towards standardization, but substantial methodological diversity persists.

In situ studies face an additional challenge related to particle size, since the smallest particle size reported in the literature rarely reflects the true sample composition. Sequential filtration during sample processing systematically removes the smallest size fractions before they ever reach the detector. Moreover, the choice of detection technique, which depends heavily on particle size, can lead to systematic over/undercounting. As a result, two critical issues emerge: the actual amount of plastic present is frequently underestimated, and the reported minimum particle size often does not correspond to the true smallest particles in the original sample. As reviewed by Zhang et al. [98], MP concentrations in tap water are generally reported to be lower than in bottled water, a discrepancy attributable to methodological limitations rather than true contamination differences. μ-FTIR, the most widely used technique, cannot reliably detect particles below 3 μm, whereas μ-Raman resolves particles well below this threshold. One of the parameters to consider in order to avoid these problems is the ability to distinguish between different points within a sample, also called spatial resolution. Table 3 shows the spatial resolution associated with different methods.

### 3.1. Fluorescence

In fluorescence detection, an excitation source at a specific wavelength illuminates the sample, which absorbs the incident radiation and re-emits it at a longer wavelength; a detector then captures this Stokes-shifted emission. Virgin PE and PP show negligible background fluorescence, whereas PS, PET, PC, and PMMA exhibit intense blue-to-green autofluorescence (400–550 nm) even without staining.

Environmental weathering and additives further enhance this effect: photo-oxidized polymers and plasticized PVC or polyurethane (PUR) can emit strongly in the yellow–orange region, overlapping with common excitation wavelengths (405 nm or 488 nm) and causing false positives [110,111,112,113]. Table 4 summarizes the autofluorescence properties of some widely used polymers, including their typical emission ranges.

**Figure 5:**
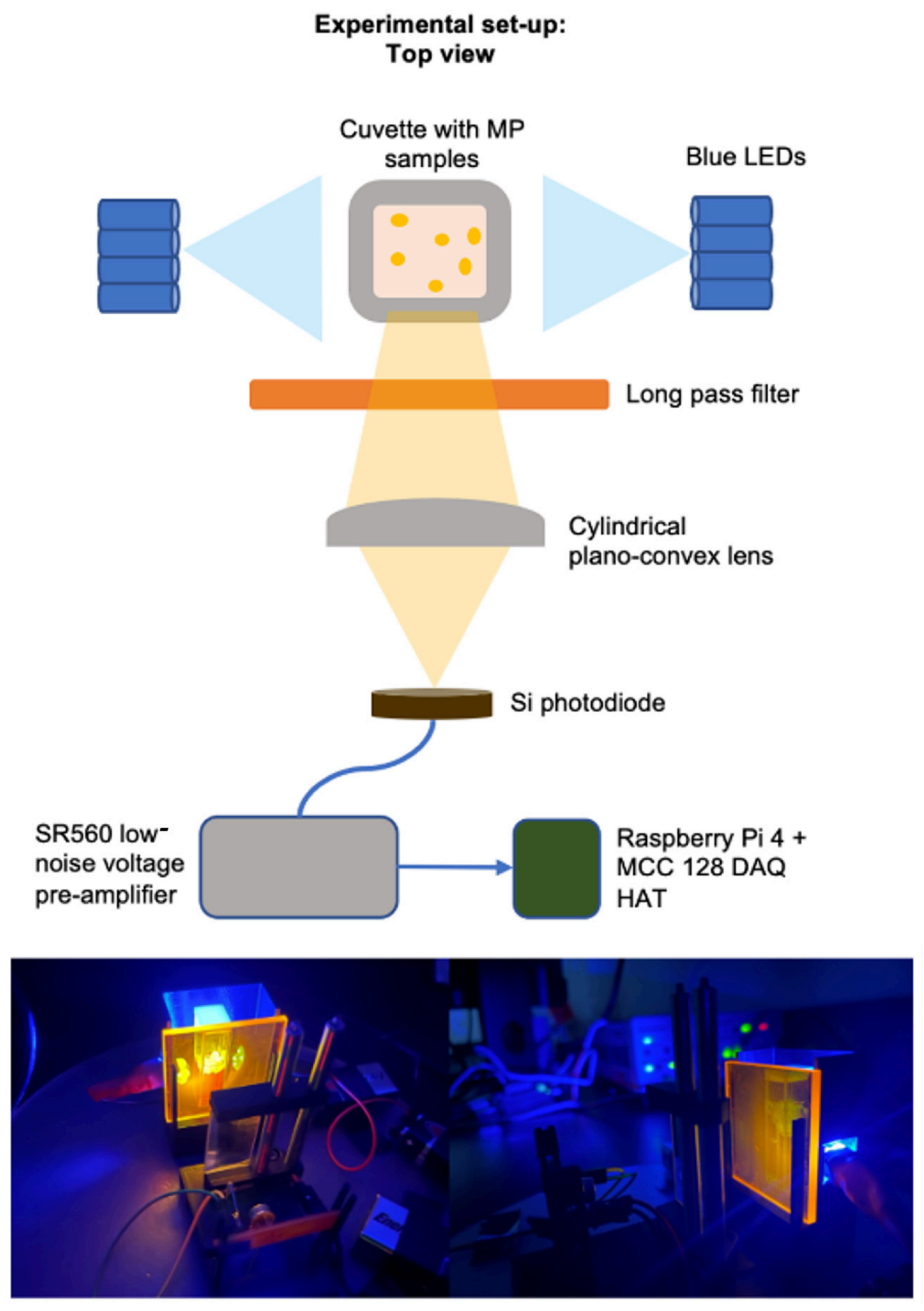
Blue light excites MPs in a cuvette, causing them to fluoresce. Fluorescent light passes through a long-pass filter and is then focused by a plano-convex lens into a photodiode receptor [123,125].

One of the most widely used fluorescence-based approaches for MP identification relies on dyes such as Nile Red, which selectively binds to plastic surfaces, rendering them brightly fluorescent upon excitation, although some natural organic particles may also exhibit partial staining, particularly in the absence of adequate pretreatment. This selective binding enables rapid visual discrimination of MPs in complex environmental samples, with reliable detection down to particle sizes of 1–20 μm, depending on the polymer type and matrix complexity [122]. The use of fluorescent dyes, particularly Nile Red, largely overcomes these issues by enabling spectral discrimination between plastics and natural organic matter. Polyolefins such as PE and PP typically emit yellow–orange fluorescence (580–620 nm), while more polar polymers such as PS, PET, and PUR shift to deeper red (640–660 nm). Although this deeper emission partially overlaps with the autofluorescence of natural compounds such as chlorophyll *a* (≥650 nm), the yellow–orange signal of polyolefins remains well separated from the biological background, enabling reliable visual and instrumental differentiation. This also allows semi-quantitative polymer class discrimination based on emission wavelength. However, this method can generate false positives due to a lack of selectivity. Nile Red may bind to residual organic matter if not fully removed via pretreatments like enzymatic or oxidative digestion. Studies have shown that Nile Red alone, without adequate organic matter removal, can lead to substantial overestimation of MP counts, in some cases up to 100%, due to the costaining of biological material [123]. S. Prasad [124] developed a low-cost optical method for MP detection and polymer identification using Nile Red fluorescent staining. Fluorescence spectroscopy enabled the grouping of common polymers into three emission classes: PE/PP, PS/PVC, and nylon/PUR/polyester. A low-cost imaging system (<USD 450) using LEDs and long-pass filters was developed (Figure 5). Low-cost photodiode sensors showed a linear response in liquid samples, demonstrating potential for portable, automated MP monitoring with future integration of machine learning.

Staining efficiency varies with polymer type and weathering state. Complementary dyes such as Safranine T and Rhodamine B further improve specificity for polymer types [110,122,125,126,127]. Table 5 presents some of the most effective and widely used dyes for staining polymers in fluorescence microscopy, along with their optimal excitation/emission wavelengths.

These staining approaches have transformed fluorescence microscopy into a cost-effective and high-throughput technique for detecting and provisionally identifying MPs down to a few micrometers in complex environmental samples [127].

#### Laser-Induced Fluorescence

Laser-induced fluorescence (LIF) is a rapid, non-destructive optical technique for the detection of MPs in complex environmental matrices. Unlike conventional fluorescence techniques that use broad-spectrum lamps and excite multiple substances simultaneously, LIF employs a laser as a highly intense and monochromatic excitation source. The narrow excitation band suppresses co-excitation of natural organic matter, algae, and sediment particles that would otherwise dominate broadband fluorescence measurements.

A 1D-CNN applied to 405 nm LIF spectra classified six marine MP types (PE, PP, PS, PET, PVC, PA) with 97.5% accuracy [132], while a two-step machine learning approach distinguished microplastics from other organic matter with 97.6% accuracy and identified the polymer type among four plastics (PE, PP, PS, PET) with 88.3% accuracy in seawater [133]. In another study, LIF spectra processed by PCA and random forest algorithms achieved 99.7% identification accuracy for mixed MP samples, highlighting the technique’s advantages in analysis speed (<1 s per particle) and cost over conventional FTIR/Raman spectroscopy for large-scale field monitoring [134]. Fluorescence lifetime imaging microscopy (FLIM), a time-resolved variant of LIF, was used to generate distinctive phasor fingerprints of unstained MPs in soil and seawater, achieving very low detection limits and demonstrating that lifetime-based methods substantially reduce false positives caused by biological material [112].

Techniques such as LIF and autofluorescence analysis bypass the limitations of staining dyes by exploiting the polymer’s intrinsic fluorescence, enabling dye-free, real-time detection [112]. By examining emission spectra and fluorescence lifetimes, it is possible to establish unique spectral fingerprints for polymers even in complex matrices like seawater or aerosols [128]. When integrated with machine learning algorithms, these approaches achieve > 90% accuracy in classifying MP types and distinguishing them from biological interferents, enabling automated, in situ chemical identification [133]. FLIM and autofluorescence analysis represent promising frontiers for achieving chemical identification directly from fluorescence signals via detailed examination of emission spectra and lifetimes (typically 1–10 ns for common polymers) [112,135]. FLIM’s phasor analysis, for instance, maps lifetime distributions to differentiate MPs from natural particles even at sub-micrometer scales [135]. Table 6 summarizes representative fluorescence-based applications for MP analysis in complex matrices, encompassing both dye-staining and label-free approaches.

### 3.2. Infrared-Based Techniques

IR spectroscopy is a widely used analytical technique that analyzes the interaction of IR radiation with matter, providing information about molecular vibrations. In traditional dispersive IR spectroscopy, IR radiation (2.5–25 μm) passes through the sample, and a monochromator sequentially scans the wavelengths. Absorption occurs when the frequency of the radiation matches the natural frequency of a molecular vibration, producing an absorption spectrum. Today, most IR spectrometers employ FTIR spectroscopy. Broadband IR radiation passes through (or is reflected from) the sample and is directed into an interferometer, which generates an interferogram. The absorption or transmittance spectrum is then obtained by applying a Fourier transform to the interferogram.

FTIR spectroscopy of aqueous samples in microfluidic systems must account for the extremely strong absorption of liquid water in the mid-IR region. Above 2 μm, where the fundamental vibrational modes of most organic and biomolecular functional groups are located, the absorption coefficient of water is so high that the IR beam intensity is attenuated by factors of roughly 100–1000 per centimeter of the optical path length. In conventional transmission cells with path lengths of hundreds of micrometers to millimeters, this results in near-complete extinction of the beam and renders standard transmission measurements impractical. In microfluidic devices, optical path lengths are typically restricted to 10–200 μm. Although such short path lengths can partially mitigate water absorption and enable transmission measurements in certain cases, the available signal remains very low, and sensitivity is severely compromised compared to non-aqueous systems. For this reason, attenuated total reflectance (ATR)–FTIR, in which the evanescent wave penetrates only 1–5 μm into the aqueous sample, has become the most widely adopted configuration for microfluidic mid-IR spectroscopy, as it effectively bypasses bulk water absorption while preserving access to the fingerprint region.

Transmission and specular reflectance FTIR differ in spectral quality, information depth, and practical applicability for polymer analysis. Transmission mode provides high SNR, flat baselines, and quantitatively reliable absorbance spectra when sample thickness is kept below 100–200 μm, allowing characterization of the bulk material [137,138]. However, strongly absorbing bands or thick/opaque samples lead to saturation or complete beam extinction. The IR beam only penetrates a few micrometers into the particle surface (wavelength-dependent), compared with the bulk characterization achieved in transmission [3]. Commercial reflectance accessories usually yield a larger beam focus (1–5 mm diameter) compared with transmission μ-spectrometers (50–200 μm), reducing lateral spatial resolution. Surface roughness or particulate morphology induces scattering and refractive index anomalies, which distort band shapes and positions. Consequently, specular reflectance is less suitable for routine bulk identification of homogeneous polymers but remains useful for surface-specific analysis of samples too thick for transmission.

FPA-based μ-FTIR imaging (FPA—focal plane array) dramatically accelerates data acquisition compared with single-point mapping, reducing measurement times from hours to minutes for areas containing thousands of particles [101]. Minimum detectable particle sizes are typically 10–20 μm in transmission or transflection mode and 20–50 μm in pure external reflectance, although sub-10 μm particles can be identified under optimal conditions with high-brightness synchrotron sources or quantum cascade laser illumination [139,140,141].

#### 3.2.1. Attenuated Total Reflection

In ATR, a high-refractive-index crystal is placed in direct contact with the sample. The IR beam enters the crystal and undergoes multiple total internal reflections, generating an evanescent field at the crystal surface that penetrates a few micrometers into the sample. The attenuated radiation emerging from the crystal carries the absorption signature of the sample and is directed to the detector. The main limitations are the shallow penetration depth of the evanescent field (typically a few micrometers, depending on the crystal and wavelength) and the requirement for good physical contact between the sample and the crystal. The choice of ATR crystal is also critical, as it must be compatible with the sample to prevent dissolution or chemical degradation upon contact. A higher angle of incidence increases the penetration depth of the evanescent field; however, angles too close to the critical angle can cause band distortion and reduced spectral quality.

The classic ATR allows only the manual analysis of manageable particles and struggles with particles < 100 μm without specialized micro-optics [3]. Othman et al. [142] successfully identified PMMA particles measuring 6 μm and 10 μm, as well as PS particles measuring 20 μm, using ATR in a benchtop configuration. Later, they developed a similar, portable, and miniaturized system for the wavelength range of 1350–4500 nm. Compact devices of this type are already widely available in academic settings, such as the equipment described in [143]. Achieving contact between very fine particles and the crystal requires significant skill and practice on the technician’s part [63]. This also means that automation is hard to achieve because particles stick to the crystal [144].

**Figure 6:**
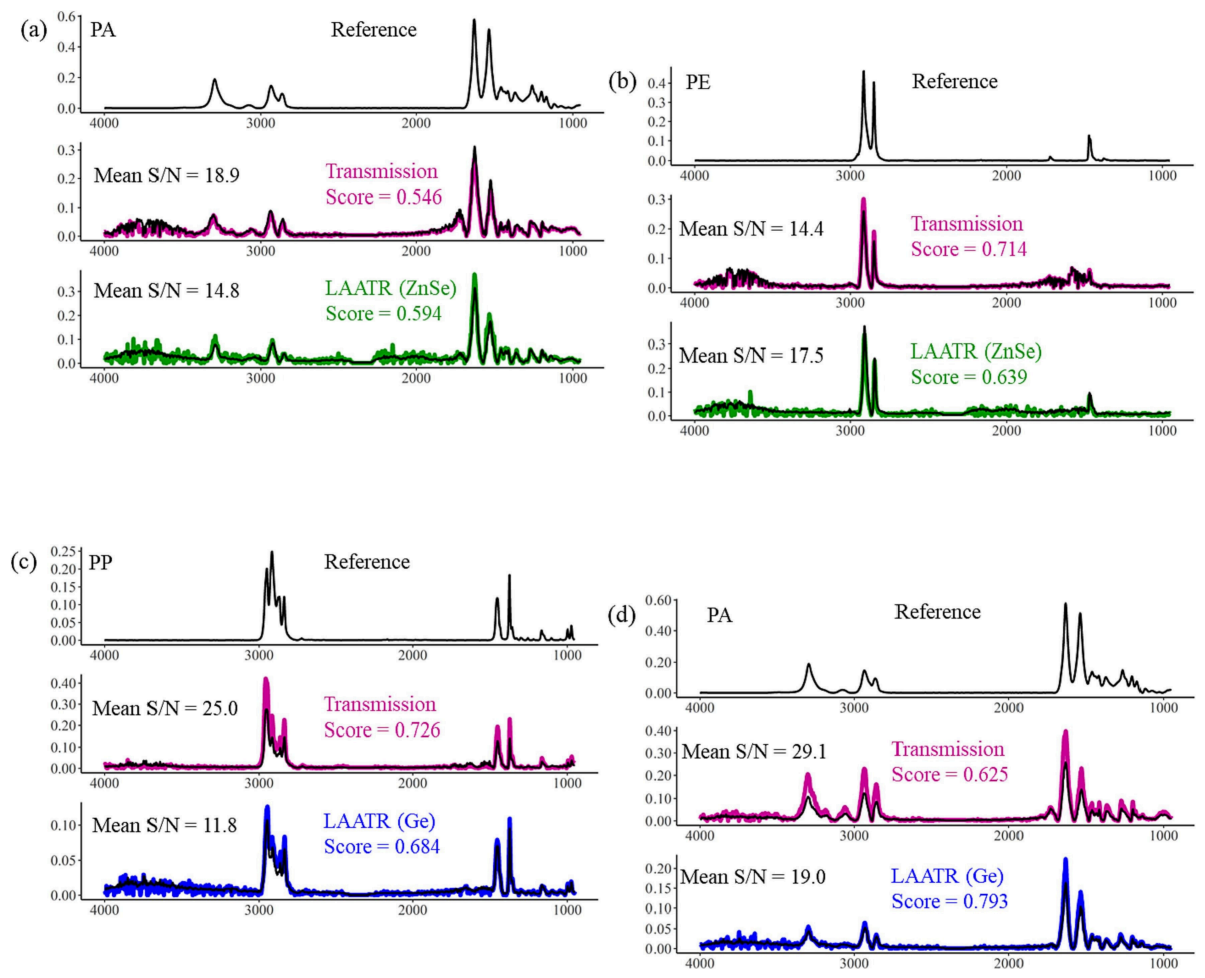
LAATR-FTIR spectra of (**a**) PA and (**b**) PE from MPs detected by transmission mode and LAATR with ZnSe crystal; representative spectra of (**c**) PP and (**d**) PA from MPs detected by transmission mode and LAATR with a Ge crystal. Colored spectra refer to max score spectra, and black spectra refer to average spectra; pink-colored spectra refer to spectra from transmission, green-colored spectra refer to spectra from the ZnSe unit; blue-colored spectra refer to the Ge unit. Spectra in the same figure come from the same MPs in different modes [147].

Recent studies demonstrated the use of ATR-FTIR for real-time chemical characterization of microfluidic streams [145]. M. Pousti et al. [146] designed an automated system capable of achieving high-resolution analysis of bacterial biofilms growing inside microfluidic devices. Y. Liu et al. [147] employed large-area attenuated total reflectance–FTIR (LATR-FTIR) to investigate smaller MP particles. They demonstrated the ability to detect and characterize reference MPs down to 1.3 μm using a germanium crystal, whose higher refractive index reduces the evanescent wave penetration depth, thereby minimizing water background signals and improving spatial resolution and sensitivity for small particles (Figure 6). This approach overcomes many of the spatial resolution constraints typical of conventional μ-FTIR. Another promising IR technique is laser direct infrared spectroscopy (LDIR). This method combines a tunable quantum cascade laser with rapid scanning and focal plane array detection, allowing fast chemical mapping and identification of microplastics directly on filter surfaces. Unlike conventional FTIR microscopy, LDIR significantly reduces analysis time while maintaining good spatial resolution and high identification accuracy, making it particularly suitable for processing large numbers of environmental samples with minimal manual intervention.

Table 7 provides an overview of representative FTIR and LDIR applications for MP analysis, illustrating the range of matrices, polymer types, and detection limits reported in the literature.

#### 3.2.2. Optical Photothermal Infrared Spectroscopy

**Figure 7:**
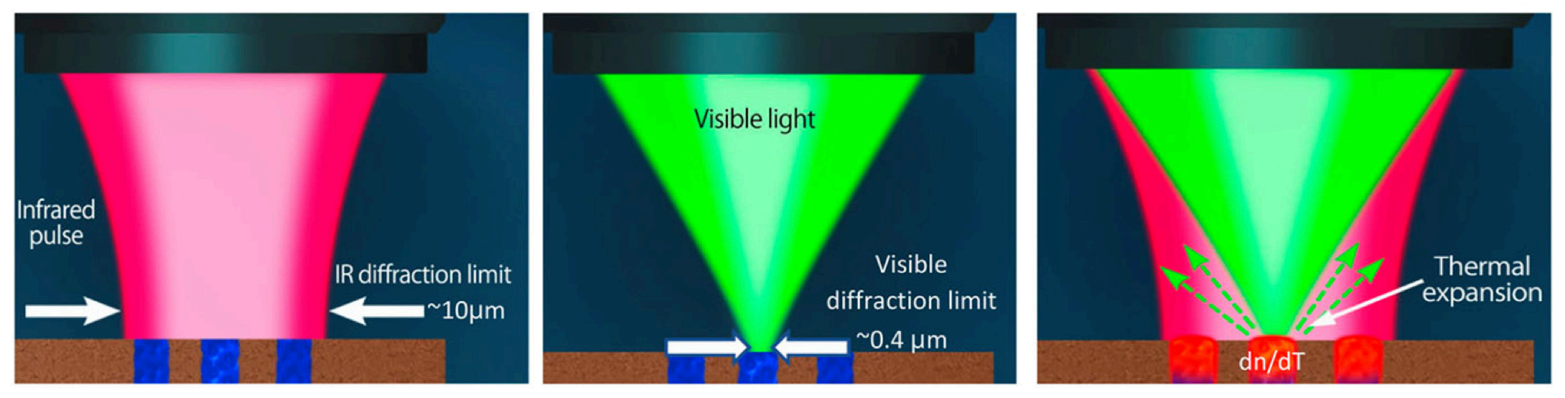
OPTIR working principle [153].

The optical photothermal IR (OPTIR) spectroscopy method employs a pulsed IR pump beam. When the frequency of this beam matches the characteristic molecular vibrational frequency of the material, energy absorption occurs. This localized absorption induces a rapid thermoelastic expansion followed by heat dissipation. The resulting thermal expansion of the material modulates the reflection or transmission of a second visible beam (probe beam), which is focused on the same spot (Figure 7). The change in the optical signal is directly proportional to the amount of IR radiation absorbed. OPTIR is a nano-IR technique that surpasses the diffraction limit associated with conventional IR spectroscopic methods, such as FTIR. This advantage arises from the fact that the spatial resolution of OPTIR is determined by the wavelength of the visible probe beam rather than by the IR excitation wavelength, enabling analysis at the nanometric scale. In the case of OPTIR combined with Raman spectroscopy, the probe beam serves a dual function: it is used both to detect thermoelastic expansion and to collect the Raman signal.

A recent study applied OPTIR to MPs in biosolids and showed that it can resolve fine fibers (2 μm wide) and small particles (5 μm), reducing artifacts associated with particle morphology [154]. In another application, OPTIR was used to detect and map nanoplastics (200 nm) in mammalian tissues, confirming the method’s capability for chemical imaging in complex biological samples [155]. A recent study using computer-controlled OPTIR coupled with Raman spectroscopy was able to identify MPs in atmospheric aerosol samples, including particles down to 0.8 μm in diameter [156].

#### 3.2.3. Hyperspectral Imaging

**Figure 8:**
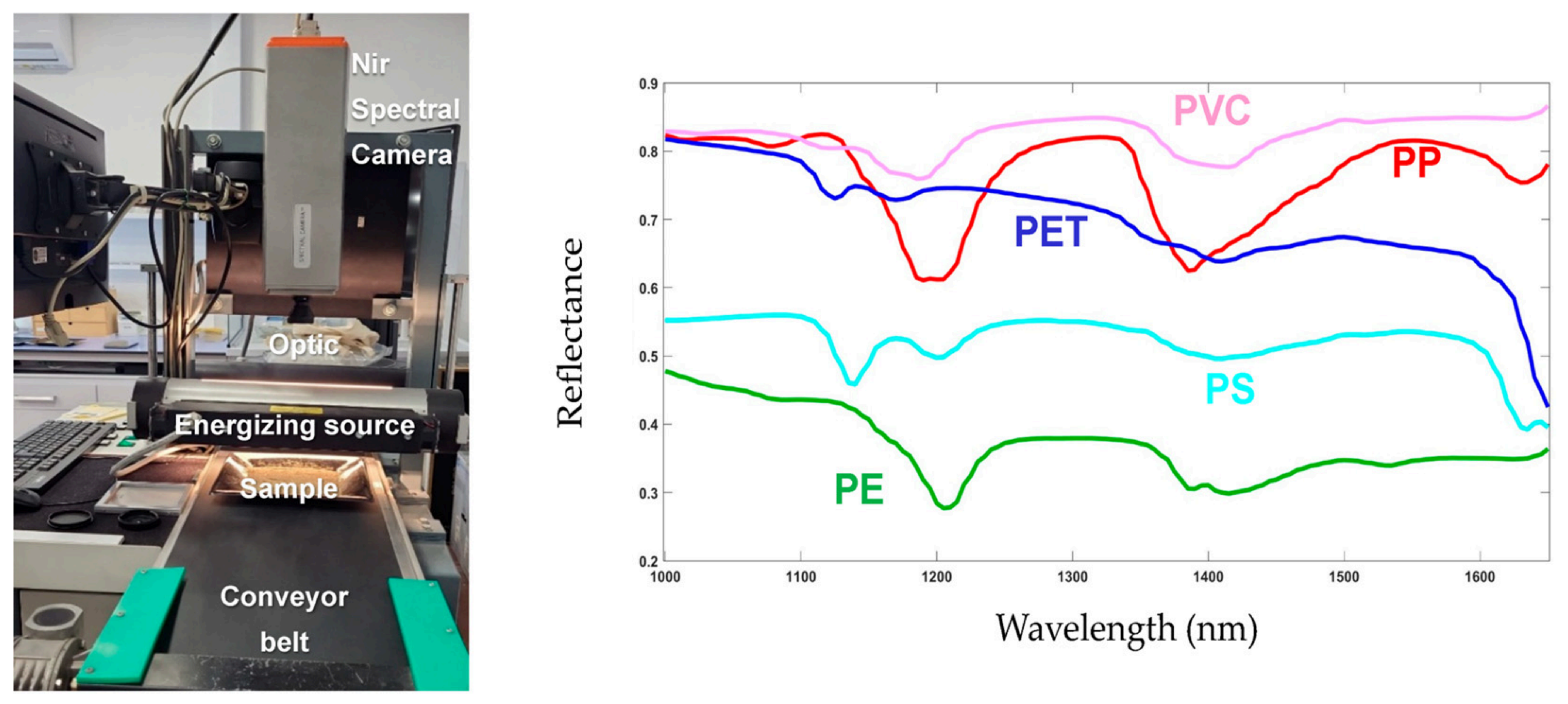
HSI acquisition system (**left**) and raw reflectance (**right**) [157].

A hyperspectral image (HSI) captures data in three dimensions, forming a hyperspectral data cube composed of two spatial dimensions (x, y) and one spectral dimension (λ). Each pixel contains a full spectral signature that describes how the material at that location reflects, absorbs, or emits light across a continuous range of wavelengths. Figure 8 illustrates an NIR-HSI acquisition system and the acquired spectra of five polymers. Because the spectral bands are narrow and contiguous, hyperspectral imaging enables highly detailed chemical and material discrimination, allowing subtle compositional differences to be detected with high spatial and spectral resolution. An overview of some HSI-based applications can be found in Table 8.

### 3.3. Raman Spectroscopy

The Raman effect refers to the inelastic scattering of light upon interaction with molecular vibrations of a sample. When a laser is directed at a sample, most of its energy is reflected in the same wavelength as the excitation, while a smaller fraction is scattered at a wavelength different from that of the excitation wavelength. This scattered radiation is known as Raman scattering. The molecular vibrations that give rise to Raman scattering are specific to each substance, providing unique insights into the composition of the sample.

In contrast to IR spectroscopy, where O–H stretching bands are extremely intense and often mask other spectral features, these vibrations are inherently weak in Raman spectroscopy. This characteristic greatly reduces water interference, making Raman particularly suitable for the analysis of aqueous samples, including biological fluids and in vivo measurements. Nevertheless, one of the major limitations of conventional Raman spectroscopy is its extreme sensitivity to sample fluorescence, which can completely overwhelm the much weaker Raman signal (typically 10^−6^ to 10^−10^ of the excitation intensity). Fluorescence occurs when the excitation wavelength overlaps with the electronic absorption bands of the sample, promoting molecules to excited electronic states that subsequently relax radiatively on a nanosecond timescale, producing broad, intense emission that obscures the sharp Raman bands.

Although the Raman spectrum of a material is independent of the excitation wavelength, allowing the same sample to be identified with different laser wavelengths, the choice of wavelength significantly impacts measurement conditions. As wavelength increases, photon energy decreases, and higher laser power is required to achieve adequate signal intensity. At longer wavelengths, especially in the near-IR (NIR) and IR ranges, greater power delivery increases sample heating due to absorption. Conversely, shorter excitation wavelengths raise the risk of photodegradation or burning. To mitigate polymer damage, strategies include rotating sample holders [160], covering the sample with Raman inert materials, cooling with gas [161] or increasing the microfluidic channel cross-section for better heat dissipation [162].

One of the major challenges in Raman spectroscopy is fluorescence interference, which can overwhelm the weak Raman signal. Deep-ultraviolet (DUV) excitation (<266 nm) effectively suppresses fluorescence. Most organic and biological molecules emit above 260 nm, avoiding spectral overlap, while the higher-energy scattered photons shift Raman bands to higher wavenumbers (>2000 cm^−1^), further separating them from residual emission [163,164]. A more accessible alternative is NIR excitation (typically 785 nm or 1064 nm), where the photon energy falls below the electronic transitions of most fluorophores, reducing fluorescence by orders of magnitude. The trade-off is the λ^−4^ dependence of Raman intensity, which reduces Raman scattering efficiency at longer wavelengths, resulting in poorer signal-to-noise ratios even before residual fluorescence is considered [63]. FT-Raman at 1064 nm nearly eliminates fluorescence but suffers from low sensitivity and higher thermal noise.

Advanced techniques further address fluorescence; for example, shifted excitation Raman difference spectroscopy (SERDS) and time-gated detection exploit the vast difference in lifetimes between Raman scattering (<1 ps) and fluorescence (nanoseconds). Using picosecond pulsed lasers and time-gated detectors, Raman photons can be selectively collected before fluorescence emerges [165,166].

Compared to FTIR, Raman spectroscopy offers two key advantages for MP analysis, namely, minimal water interference, enabling identification in large aqueous volumes [167], and access to low wavenumber regions (<600 cm^−1^), allowing simultaneous characterization of organic polymers and inorganic pigments/fillers [63].

Despite these strengths, conventional Raman suffers from an intrinsically low scattering cross-section, yielding weak signals that demand long acquisition times (seconds to minutes) or high laser power (>100 mW) [168]. This has historically limited its use in portable or field applications. To overcome these constraints, enhanced techniques such as surface-enhanced Raman scattering (SERS), resonance Raman, coherent anti-Stokes Raman scattering (CARS), and stimulated Raman scattering (SRS) provide signal amplification of 10^3^–10^14^, enabling sub-micron spatial resolution and even single-molecule sensitivity in complex matrices [169,170]. Parallel advances in diode laser miniaturization, high-throughput spectrometer design, and compact CCD/CMOS detectors have produced handheld instruments light enough for single-operator field deployment [171,172].

Micro-Raman spectroscopy (μ-Raman) or confocal Raman microscopy employs an optical microscope to focus the excitation laser into a diffraction-limited spot (typically <1 μm in lateral diameter and a few micrometers in depth), thereby providing significantly enhanced spatial resolution and sensitivity compared to conventional Raman spectroscopy. This enables the analysis of microscopic or highly dilute samples and facilitates 2D and 3D chemical mapping. However, its performance can be compromised on rough or irregularly shaped surfaces due to defocusing and increased stray light scattering. The instrumentation is considerably more complex and expensive than conventional Raman systems, primarily because of the integration of a high numerical aperture objective, confocal pinhole, and precise scanning stages.

Resonance Raman spectroscopy (RRS) uses an excitation wavelength that deliberately matches an electronic absorption band of the target molecule. This resonance condition selectively boosts the Raman scattering intensity of vibrational modes coupled to that electronic transition by factors of 10^3^ to 10^6^, dramatically increasing sensitivity and enabling detection limits in the nanomolar to micromolar range, even in complex or highly interfering matrices. At the same time, the Raman signal usually overwhelms the broader, red-shifted fluorescence background, effectively suppressing it. The main limitation of RRS is the need for tuneable lasers or multiple discrete laser lines precisely aligned with the analyte’s absorption profile, which significantly increases both instrument cost and experimental complexity compared to conventional fixed wavelength Raman or μ-Raman systems.

Raman hyperspectral mapping extends point-by-point μ-Raman analysis to 2D or 3D datasets by scanning the laser across a defined sample area and recording a full Raman spectrum at every pixel. Although traditional point-by-point mapping provides excellent spectral quality, it is notoriously slow. For instance, acquiring a 100 × 100 μm^2^ map with 1 μm resolution and 1 s integration time per spectrum requires several hours of continuous measurement [173]. This becomes a serious problem when studying particles or features smaller than 10 μm: the weak Raman scattering and interference from the surrounding matrix make manual positioning difficult and prevent statistically reliable analysis of hundreds or thousands of individual particles. Modern systems overcome these issues with precise motorized stages, fast detectors, and full automation. Newer techniques (line illumination, wide-field imaging, multifocus) have cut acquisition times from hours to minutes or seconds without losing 1 μm resolution [174,175].

#### 3.3.1. Shifted Excitation Raman Difference Spectroscopy

The primary objective of the SERDS method is to suppress the background fluorescence that typically obscures the Raman signal in biological and polymeric samples. Fluorescence produces a broad band emission that is largely independent of the excitation wavelength, whereas the Raman signal appears as a wavelength shift relative to the excitation source. SERDS acquires two spectra using slightly shifted excitation wavelengths and computes their difference. This differential approach effectively cancels the fluorescence background while preserving the Raman features of interest.

#### 3.3.2. Surface-Enhanced Raman Spectroscopy

SERS is a spectroscopic technique that amplifies the inherently weak Raman scattering signal of molecules when they are adsorbed onto or in proximity to nanostructured noble metal surfaces. Excitation radiation interacts with metallic nanostructures, leading to the collective oscillation of the metal’s free electrons. This phenomenon, known as localized surface plasmon resonance (LSPR), generates a highly intensified local electromagnetic field near the nanostructure’s surface. The resulting Raman signal intensity can be increased by a factor of 10^6^ to 10^11^ in typical configurations, with values approaching 10^14^ reported under optimal single-molecule conditions.

**Figure 9:**
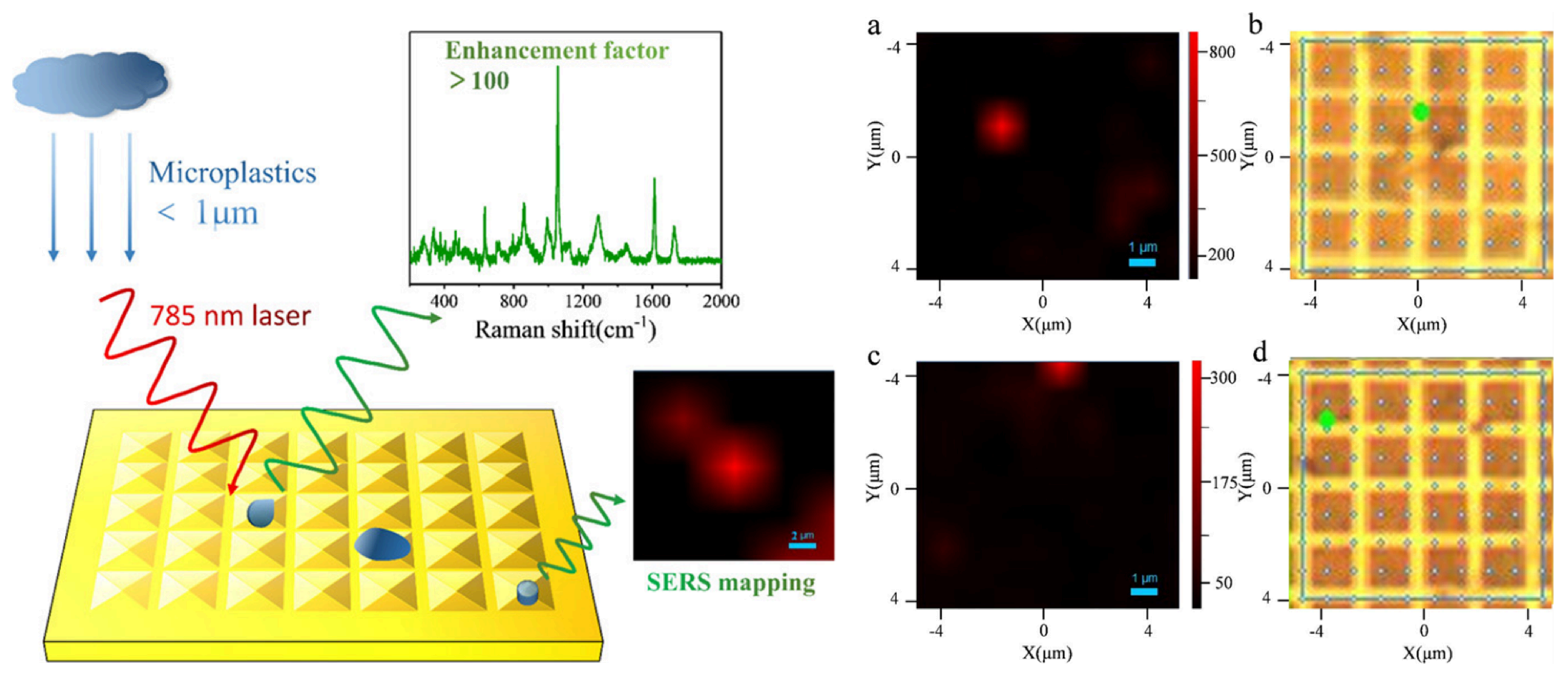
(**a**,**b**) Raman mapping image (**a**) and optical, bright-field microscopic image (**b**) of PET particles on Klarite. (**c**,**d**) Raman mapping image (**c**) and microscopic image (**d**) of PS particles on Klarite. Scale bar: 2 μm. The green spot in (**b**,**d**) is the laser focus point, while the white spots are the sampling points [178].

In the context of MP analysis, SERS is typically performed using Ag or Au nanoparticles, or nanostructured substrates, that aggregate around or are deposited onto the target polymer particles. Reported LODs vary considerably depending on particle size, polymer type, and substrate geometry. Chaisrikhwun et al. [176] achieved 0.1 μg·mL^−1^ for PS nanoplastics using a gold-sputtered substrate combined with a coffee-ring pre-concentration step, while Caldwell et al. [177] reported LODs of 1.25 μg·mL^−1^ for 33 nm PS and 5 μg·mL^−1^ for 36 nm PET using gold nanostar-based substrates in aqueous matrices. Xu et al. [178] demonstrated detection of environmental NPs using a Klarite nanostructured Au substrate that generates localized hotspots capable of enhancing the Raman signal by up to two orders of magnitude for polystyrene analytes (Figure 9). Combined with Raman mapping, this approach showed potential for faster, field-applicable environmental monitoring.

#### 3.3.3. Tip-Enhanced Raman Spectroscopy

When a sample is smaller than the diameter of the laser spot, the resulting Raman spectrum will include contributions not only from the sample itself but also from the surrounding material. To overcome this limitation, Tip-Enhanced Raman Spectroscopy (TERS) employs an ultrasharp metallic tip typically coated with a plasmonic metal such as gold or silver positioned only a few nanometers above the sample surface. When the laser illuminates the tip, a localized surface plasmon resonance is generated, producing an intense electromagnetic field enhancement (10^3^–10^7^) at the tip–sample junction. The field confinement at the tip apex restricts signal collection to a nanometer-scale volume, breaking the diffraction limit and ensuring that the acquired spectrum reflects the local chemistry directly beneath the tip rather than an averaged bulk response. Furthermore, by raster scanning the tip across the surface, it is possible to obtain high-resolution chemical maps of the sample.

One notable drawback of TERS is tip degradation. Silver tips tend to oxidize rapidly, while gold tips provide weaker field enhancement [105]. TERS can be implemented in two configurations: transmission mode for transparent samples and reflection mode for opaque samples. Despite its potential, the instrumentation remains highly complex and costly [105].

#### 3.3.4. Coherent Anti-Stokes Raman Scattering

CARS is a nonlinear optical technique that produces a coherent, high-intensity signal. It relies on the interaction of two waves, specifically a pump beam and a lower-energy Stokes beam. When the frequency difference between these two beams matches the molecule’s specific Raman vibrational frequency, the molecular ensemble is driven into phase coherence, meaning that all molecules within the laser focus vibrate synchronously. Following this excitation process, a third photon interacts with the coherent vibration, generating a new photon at an anti-Stokes frequency. This signal appears at a shorter wavelength than the excitation beams, making it easier to detect due to the absence of a fluorescence background. The CARS signal is typically 10^3^–10^5^ times stronger than spontaneous Raman scattering. These properties make CARS the Raman variant best matched to the demands of continuous-flow MP detection, where acquisition times must be short enough to characterize particles as they transit the probe volume. The principal artifact in CARS is a non-resonant background, a spectrally flat four-wave-mixing signal from the solvent and substrate that distorts peak shapes and undermines quantitative band assignments unless actively suppressed [179,180]. Beyond background suppression requirements, the use of two pulsed laser sources introduces greater complexity in alignment and calibration, as well as higher system costs [181].

**Figure 10:**
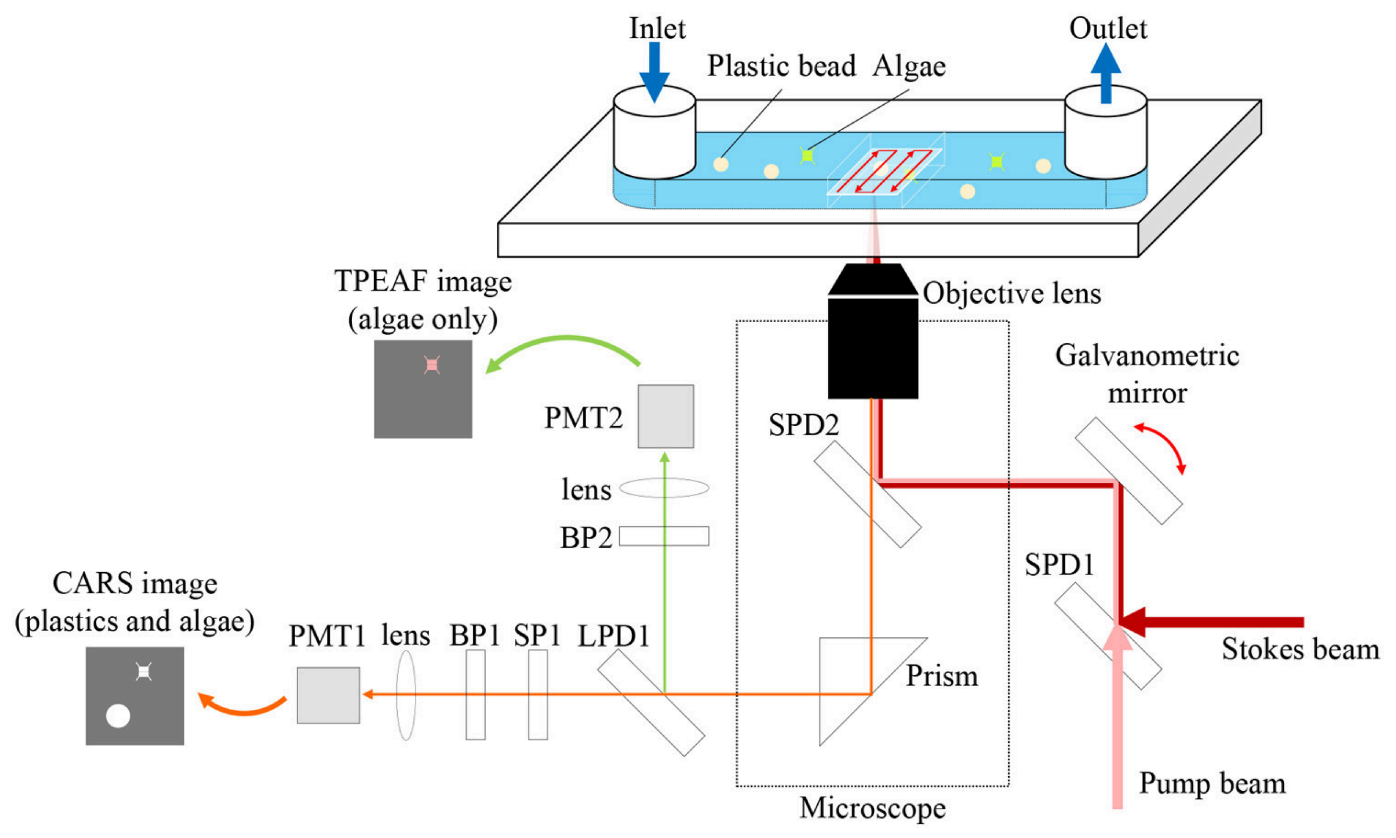
CARS + 2-photon-excited autofluorescence experimental setup. SPD, short-pass dichroic beam splitter; LPD, long-pass dichroic beam splitter; BP, band-pass filter; SP, short-pass filter and PMT, photomultiplier tube [182].

Multimodal CARS combined with Two-Photon Excited Autofluorescence (TPEAF) enables rapid, high-resolution detection and classification of MPs and organic particles in continuous flow (Figure 10) [182]. The method successfully identified MPs with sizes ranging from tens to several hundred micrometers while simultaneously distinguishing them from organic particles via autofluorescence. This approach allowed real-time monitoring of MPs in aqueous flow without requiring filtration, separation, or other preparatory steps, offering a high-throughput and non-destructive alternative to conventional spectroscopy-based techniques. Table 9 compiles representative Raman-based applications for MP identification, spanning conventional μ-Raman, SERS, CARS, and SRS across a wide range of matrices, particle sizes, and excitation wavelengths.

#### 3.3.5. Raman Comparative Analysis and In Situ Viability

Raman scattering offers notable advantages in aqueous environments due to its insensitivity to water absorption. However, its direct in situ application is fundamentally hindered by low signal-to-noise ratios and significant environmental background interference. Conventional Raman signals require extended acquisition times, making the chemical analysis of continuously flowing particles highly impractical. Consequently, successful on-chip integration necessitates upstream microfluidic manipulation, such as acoustic or optical trapping, to physically immobilize target particles during spectral acquisition.

Furthermore, environmental samples contain complex nonplastic interferents, including organic debris and biological matter, which generate intense autofluorescence that can completely obscure the weak Raman signature. Therefore, Raman spectroscopy cannot operate effectively as an isolated sensor in raw environmental matrices. To achieve reliable MP identification, it must function within a synergistic platform where upstream separation modules selectively isolate polymers from fluorescent interferents. When paired with fluorescence suppression techniques like NIR excitation or SERDS, μ-Raman emerges as a highly robust identification tool. Advanced variants such as CARS and SERS offer transformative signal amplification yet currently remain high-resolution laboratory benchmarks rather than field-deployable continuous-flow solutions.

## 4. Data Analysis and Machine Learning Approaches

Environmental MP spectra are rarely clean. Weathering shifts peak positions, biofouling introduces fluorescent backgrounds, and particles composed of polymer blends or carrying surface additives produce composite signatures that diverge substantially from pristine library references [194]. Direct library matching therefore tends to underperform on field-collected material, and its failure rate increases with the degree of environmental aging to which samples have been subjected. Machine learning classifiers address this limitation by learning the spectral variance characteristic of real degradation states, rather than relying on fixed spectral templates. By operating on selected wavelengths rather than full spectra, these algorithms also reduce computational cost and improve robustness to the diverse additive compositions present in commercial plastics [195]. The following subsections describe the main algorithmic families reported in the MP identification literature, with emphasis on their specific performance in the studies compiled in Table 10.

### 4.1. Principal Component Analysis and Linear Discriminant Analysis

In MP spectral analysis, principal component analysis (PCA) serves primarily as a dimensionality-reduction step upstream of classification, rather than as a classifier in its own right. A Raman spectrum typically spans hundreds or thousands of wavenumber channels, most of which carry redundant information. PCA projects this high-dimensional space onto a reduced set of principal components (PCs) that capture the dominant sources of spectral variance, discarding noise and collinear variables. This compression improves computational efficiency and can enhance the separability of subsequent classifiers such as k-nearest neighbor (KNN), support vector machine (SVM), or random decision forest (RDF). In the context of nanoplastic identification, Luo et al. [186] extended the PCA framework into a dual-PCA approach that correlates spectral matrices directly against reference polymer scores, enabling automatic identification and 2D spatial mapping of PE, PVC, and PA6 particles at the sub-micrometer scale from μ-Raman data, a regime in which conventional library matching fails because weak scattering cross-sections produce spectra with poor SNR. The same group subsequently combined this dual-PCA step with an algebra-based spectral merging pipeline to extend coverage to ten polymer types, demonstrating the detection of nanoplastics down to 100 nm [187]. The principal limitation of PCA in this domain is that its components are mathematical constructs with no direct spectroscopic meaning, which complicates interpretation and means that environmentally relevant variance may be distributed across multiple PCs rather than isolated in one.

Linear discriminant analysis (LDA) complements PCA by incorporating class label information. Whereas PCA maximizes total variance irrespective of class membership, LDA seeks the linear combination of features that simultaneously maximizes inter-class separation and minimizes intra-class scatter. In practice, LDA is applied as a postprocessing step after PCA has reduced spectral dimensionality, with the resulting PCA + LDA pipeline exploiting the strengths of both transformations. Feng et al. [190] demonstrated this combination on μ-Raman, covering seven household polymer types, and reported overall classification accuracies exceeding 98% on standard, real, and environmentally stressed samples. Notably, the reliefF feature selection algorithm proved essential in that study for separating spectrally similar polymer pairs such as HDPE and LDPE, whose Raman bands overlap extensively. A practical constraint on LDA is its assumption of multivariate normality and equal class covariance conditions that are unlikely to hold for heterogeneous environmental samples in which particle morphology, surface contamination, and additive variability introduce non-Gaussian spectral scatter within each polymer class.

### 4.2. Random Decision Forest

RDF classifiers have found wide application in high-throughput MP imaging workflows, where the requirement to classify millions of spectra per sample prohibits computationally intensive methods. Hufnagl et al. [196] applied RDF to μ-FTIR transmission data from environmental samples, achieving true positive rates of 94–100% for PE, PP, PMMA, PAN, and PS and completing the classification of a one-million-spectrum image within minutes on a standard desktop PC. This throughput advantage reflects the inherent parallelism of ensemble tree methods: because each tree is trained on an independent random subset of the data and a random subset of spectral features, inference across a large dataset scales efficiently. RDF has also demonstrated competitive performance when coupled with fluorescence-based detection. Meng et al. [134] combined PCA with RF to process LIF spectra of marine MPs, achieving 99.7% composition identification accuracy and a mass–concentration correlation coefficient exceeding 0.99. The results were attributed in part to RF’s ability to resolve severely overlapping fluorescence signatures between PE, PP, and PS without requiring explicit fluorescence background subtraction. Similarly, Xie et al. [189] trained an RF model on μ-Raman spectra of five nanoplastic types on aluminum substrates, reaching 98.8% average accuracy and 100% specificity at particle sizes down to 360 nm, with validation on spiked tap water confirming transferability to real matrices. The principal drawback of RDF in this context is interpretability: the ensemble of hundreds of trees does not produce a transparent decision rule, which makes it difficult to identify which spectral features drive classification. This limitation matters when seeking to understand whether a model is exploiting genuine polymer-specific bands or spurious artifacts introduced by the sample matrix or substrate.

### 4.3. K-Nearest Neighbor

KNN assigns a query spectrum to the class most frequently represented among its k-nearest neighbors in feature space, without constructing an explicit decision boundary. This non-parametric behavior makes KNN sensitive to the local geometry of the spectral distribution, which can be advantageous when polymer classes occupy compact, well-separated clusters but becomes a liability in high-dimensional spaces where distance metrics lose discriminative power. For this reason, KNN is almost exclusively used in the MP literature as part of hybrid pipelines where PCA or another dimensionality-reduction step precedes classification. Feng et al. [190] included PCA + KNN as one component of a multi-model ensemble alongside PCA + LDA and MLP, with the combined approach outperforming any single classifier on environmentally stressed μ-Raman spectra. In a different spectroscopic context, Sarmiento et al. [79] evaluated KNN alongside SVM and decision trees for classifying PET MPs from EIS impedance signatures, finding that KNN achieved the highest accuracy in both pure water (86.6%) and complex environmental matrices (89.2%), the latter containing organic and inorganic interferents designed to simulate realistic field conditions. The authors attributed KNN’s advantage to its exploitation of local impedance feature similarity without imposing a global parametric boundary, a property that appears particularly relevant when the feature space is low-dimensional, as is the case for EIS-derived descriptors relative to full Raman or FTIR spectra. In high-dimensional spectral datasets, the computational cost of distance calculation over all training examples becomes a practical constraint on throughput, limiting KNN’s applicability in large-area mapping campaigns.

### 4.4. Support Vector Machine

SVM constructs the optimal separating hyperplane between classes by maximizing the margin between class boundaries and the nearest training examples. When classes are not linearly separable in the original feature space, a kernel function projects the data into a higher-dimensional space where linear separation becomes feasible, at the cost of additional hyperparameter selection. SVM has become one of the most widely benchmarked classifiers in MP spectral analysis, frequently serving as the baseline against which newer approaches are evaluated. In the EIS study by Sarmiento et al. [79], SVM underperformed KNN in complex matrices (81.2% vs. 89.2%), which the authors linked to the sensitivity of kernel-based methods to the choice of regularization parameter when training data are limited. In the μ-Raman domain, Ren et al. [191] compared SVM against a CNN on a ten-polymer dataset covering 1660 spectra across the 10–500 μm size range, finding that the CNN outperformed SVM in both accuracy (96.43% vs. lower for SVM) and robustness to spectral variability introduced by weathering and additive heterogeneity. In a laser-induced fluorescence setting, Merlemis et al. [133] applied a two-step machine learning workflow to seawater samples: the first stage separated microplastics from other organic material with 97.6% accuracy, and a second stage identified the polymer type among four plastics (PE, PP, PS, and PET) with 88.3% accuracy.

### 4.5. Artificial and Convolutional Neural Networks

Artificial and convolutional neural networks (ANNs and CNNs) learn feature representations directly from raw spectral data through successive nonlinear transformations, without requiring manual feature engineering. The 1D-CNN architecture has proven particularly well suited to spectral classification tasks because its convolutional filters operate over local wavenumber windows, learning to detect polymer-specific band patterns irrespective of their absolute position in the spectrum. Zeng et al. [197] trained a 1D-CNN on FTIR spectra of virgin and UV-aged PE, PP, and PS, reporting 100% accuracy on aged commercial MP samples, compared with 80% for a standard deep neural network and 60% for RF. The authors attributed this performance gap to the CNN’s ability to preserve raw spectral detail without additional preprocessing, which is critical when aging-induced peak broadening and baseline shifts must be treated as informative variance rather than noise. In μ-Raman workflows, Ren et al. [191] achieved 96.43% accuracy across ten polymer types with a CNN trained on 1660 spectra. Lim et al. [192] demonstrated that a CNN with tailored spectral interpolation could classify particles in the 1–10 μm range at 0.4 s exposure per particle, approximately 67 times faster than conventional full-mapping Raman spectroscopy, by learning to extract diagnostic features from short acquisitions that would be rejected as insufficient by library-matching routines. In a directly microfluidic context, Gong et al. [183] applied a CNN to on-chip μ-Raman spectra from surface seawater, achieving 93% classification accuracy across eleven polymer types with a mean AUC of 98%, outperforming SVM, RF, and a ResNet34 architecture on the same dataset.

Neural networks also appear in hybrid configurations that combine automated pre-screening with expert validation. Weber et al. [188] developed a human–machine teaming framework in which a per-polymer-class MLP pre-filters μ-Raman spectra at a low confidence threshold (0.1), flagging borderline cases for expert review. This approach achieved recall ≥ 99.4% and precision ≥ 97.1% across five polymer types on real environmental and wastewater samples. Analogously, Feng et al. [190] integrated MLP into a multi-model ensemble that combined PCA-LDA and PCA-KNN, demonstrating that MLP contributes to classification accuracy on spectra where the linear methods fail. A more recent development is transfer learning, in which a network pre-trained on large spectral corpora is fine-tuned for a specific polymer subset or environmental matrix. This approach directly addresses the scarcity of labeled training data for weathered and biofouled particles, which remains the principal data bottleneck in MP machine learning. The primary constraints on ANNs and CNNs are their data requirements. The datasets in the reviewed studies range from approximately 1200 to over 72,000 spectra [189,192] and the opacity of their learned representations, which prevents direct attribution of a classification decision to a specific spectral feature.

The methods described above differ substantially in their complexity, data requirements, and interpretability. PCA and LDA are computationally efficient and easy to interpret, but their linear nature may limit performance when spectral classes overlap significantly. KNN is intuitive and effective for small, well-defined datasets, yet it scales poorly with increasing data volume. SVM offers robust performance in high-dimensional spaces and handles overlapping classes well, at the cost of more demanding parameter tuning. RDF provides high accuracy and resilience to noisy or incomplete data through ensemble learning, though it remains a black-box model. ANNs and CNNs represent the most powerful approaches, capable of autonomously extracting complex spectral features and achieving state-of-the-art performance, but they require large training datasets and considerable computational resources and offer limited interpretability. In practice, hybrid approaches are frequently adopted to combine the strengths of dimensionality reduction with robust classification.

## 5. Conclusions

MPs persist in the environment for extended periods, posing serious concerns for both public health and the environment. Despite the complex capture of MPs, current society has the power to eliminate the use of primary MPs and capture macropolymers from the ocean before they degrade and break down into MPs. While developed countries are progressively taking measures to reduce plastic pollution, less developed countries heavily rely on the plastic industry, making it challenging to mitigate this issue entirely [198,199]. Studies tracking the proliferation of MPs in various ecosystems still face challenges related to under/overcounting and identification accuracy. Significant contributions can be made by developing in situ methodologies for MP identification and establishing continuous monitoring networks to provide a more precise and accurate description of MP proliferation. LOC devices reduce reagent consumption, instrument footprint, and operational costs and are therefore well suited to the continuous, long-duration deployments required for meaningful MP surveillance [200].

To maintain high repeatability and low cost during extended monitoring, modular LOC designs featuring easily replaceable microfluidic channels represent the most viable path to mitigating long-term fouling. Furthermore, for autonomous in situ applications, techniques requiring extensive pretreatment, reagents, or fluorescent labels are poorly suited, making direct, reagent-free spectroscopic methods the superior choice for continuous surveillance.

Many of the methods described in the literature have been extensively tested in the laboratory using commercially acquired pellets. However, MPs found in the environment present optical and physicochemical properties that are distinct from those of raw pellets. Their coloration, morphology, chemistry and optical properties are affected by the level of degradation to which they are exposed, and therefore, a laboratory method that performs well may not necessarily exhibit similar performance in situ [201].

Raman and FTIR methods, along with their variants, are the most employed techniques for the chemical identification of MPs in microfluidics. Although both μ-FTIR and μ-Raman spectroscopy are well-established for MP polymer identification, their performance characteristics differ substantially and favor complementary deployment rather than direct substitution. FTIR imaging offers faster throughput and remains reliable for particles in the 50–500 μm range. However, its practical detection limit in environmental samples falls closer to 50 μm, well above the nominal specification of 10–20 μm, leading to severe underestimation of smaller size fractions [63]. Raman imaging provides superior spatial resolution, capable of resolving particles down to 1 μm, but it is susceptible to fluorescence interference from pigments, organic contaminants, and weathered polymer surfaces, a limitation of particular relevance in environmental matrices [71]. For colored particles, neither technique alone ensures reliable chemical characterization, and a combined approach is recommended [63]. Within a microfluidic context, both techniques face additional matrix-specific constraints. In Raman spectroscopy, the characteristic spectral background of PDMS overlaps with analyte peaks across broad spectral regions, requiring dedicated subtraction strategies [57]. Autofluorescence from PMMA and other thermoplastic substrates constitutes an additional source of interference at shorter excitation wavelengths [57]. For FTIR, strong mid-infrared absorption by water limits the usable transmission path length to below 10 μm under conventional broadband illumination, a constraint fundamentally incompatible with standard microfluidic channel geometries [145]. However, these methods often rely on comparison with library spectra, leading to misidentification of various particles, primarily due to the reasons stated previously [201]. In addition, different plastic producers may add varying amounts of additives to the same plastic, leading to overlapping peaks and potential misidentification. One approach is to expand existing spectral libraries to include reference spectra of UV-aged, biofouled, and additive-rich MPs and to use these extended libraries as training data for machine learning classifiers rather than relying on direct library matching.

Adapting existing spectroscopic methods specifically for MP identification offers measurable gains in analytical performance. Targeted polymer libraries reduce false positive rates relative to full-spectrum library matching, while restricting analysis to selected wavenumber regions cuts acquisition time and computational load. At the device level, miniaturization reduces instrument footprint and reagent consumption, lowering the operational cost of continuous deployments, a critical factor for long-term in situ monitoring networks.

## Figures and Tables

**Table 3:** Spatial resolution of the most common methods.

Technique	Typical Spatial Resolution	Notes	Ref.
FTIR (transmission/reflectance, macro, no microscope objective)	5–20 μm	Diffraction-limited (λ/2NA). Resolution below 5 μm is rare without synchrotron sources.	[99]
μ-FTIR (microscope-coupled, transmission/reflectance)	3–10 μm	Diffraction-limited as above. Smaller apertures/higher NA objectives push toward the lower end. Enables sub-10 μm mapping of fibers and films.	[63,100]
FPA-FTIR	3–10 μm	Diffraction-limited resolution. Actual pixel projection on a sample can be finer, but true resolving power remains diffraction-governed, not pixel-governed.	[101]
μ-ATR-FTIR (ATR objective, e.g., Ge or diamond crystal)	3–5 μm	Higher refractive index of the ATR crystal (n ≈ 2.4–4) effectively reduces λ, improving resolution beyond conventional transmission μ-FTIR.	[102]
μ-Raman	0.2–1 μm	Achieves 250–500 nm lateral resolution with high-NA objectives and visible lasers. Sub-200 nm possible with 405 nm excitation and special objectives.	[103]
SERS	30 nm–1 μm *	Practical resolution depends on the nanostructured substrate.	[104]
TERS	1–20 nm	Sub-10 nm resolution under ultra-high vacuum and 10–20 nm in ambient conditions.	[105]
CARS	0.2–1 μm	Axial resolution significantly better (0.7–2 μm).	[106,107]
Hyperspectral imaging	15–150 μm per pixel	Resolution determined by optics, sensor pixel size, and spectral range.	[108,109]

* The range given refers to the minimum particle size detectable.

**Table 4:** Polymer autofluorescence bands.

Polymer	Autofluorescence (Experimental Conditions May Affect)	Principal Emission Range	Key Observations	Ref.
PE	Very weak	Minor emission at 450–550 nm after prolonged aging	Remains one of the least autofluorescent polymers even after extensive UV exposure and oxidation.	[114,115]
PP	Weak if virgin but increases with photo-oxidation	400–520 nm	Photo-oxidative degradation generates carbonyl groups responsible for enhanced blue–green emission.	[116]
PS	Moderate	350–460 nm	High intrinsic fluorescence from aromatic rings. Severe interference with 405 nm excitation in microscopy.	[116,117]
PET	Moderate to strong	400–550 nm	Autofluorescence intensity is linked to amorphous regions and additives.	[118]
PA6 and PA66	Moderate if virgin; strong after thermal degradation	420–580 nm	Thermal oxidation and chain scission significantly increase emission in the blue–green region.	[113,117,119]
PVC	Weak to moderate	450–650 nm	Plasticizers and thermal stabilizers contribute to autofluorescence. Signal may vary with processing.	[120]
PMMA	Moderate	390–520 nm	One of the most problematic polymers for unstained fluorescence microscopy due to intense intrinsic signal.	[57,121]
PC	Moderate	400–540 nm	Aromatic bisphenol-A backbone confers high autofluorescence, particularly under UV–blue excitation. Varies with additives.	[57]
PES	Moderate	420–580 nm	Dyed or finished fibers can exhibit drastic shifts or quenching of intrinsic autofluorescence.	[122]

**Table 5:** Principal dyes used in MP staining for fluorescence identification.

Dye	Excitation Wavelength	Emission Wavelength	Key Observations	Ref.
Nile Red	405, 465, 525 nm	469–650 nm	Universal dye. Distinguishes nonpolar polymers (PE/PP). from polar polymers (PS, PET, PA).	[122,128]
Safranine T	530 nm	580–590 nm	Excellent staining of PE and PP. Low affinity for PS.	[129,130]
Fluorescein	488 nm	519 nm	Stains PS, PE, PVC and PET universally. Weaker overall performance than Nile Red.	[130,131]
Rhodamine B	540–561 nm	580–630 nm	High affinity for PVC, PS and PUR.	[126,129]

**Table 6:** Overview of representative fluorescence-based applications for MP analysis in complex matrices.

Technique Used	Excitation Wavelength	Matrix	Polymers Analyzed	Particle Size	Performance Metrics	Ref.
Nile Red	450–510 nm	Marine sediments	PE, PP, PS, PET, PVC, PA6	Theoretical detection limit 5 μm particles tested: 0.1–0.5 mm	Recovery averaged 96.6% in coarse sand but decreased to 85–88% in fine silt, likely due to particle entrapment. Algae did not produce false positives under the conditions used; however, mineralized chitin and black carbon fragments were identified as potential interferents.	[127]
	405 nm, 465 nm and 525 nm	Beach sediment Laboratory aqueous solutions (PP pellets)	PP, PE, PS, PET, PUR, PVC, Nylon	NR	Fluorescence emission spectra provided a preliminary basis for distinguishing between NR-stained polymer types. Field trial results showed 80% correct identification of PP samples and 100% of PE samples. PP and PE could not be fully distinguished from each other by spectral data alone. A proof-of-concept photodiode-based detection system showed a linear response to MP concentration in liquid samples.	[123]
	405 nm, 465 nm, 525 nm	Solid fragments	PE, PP, PS, PVC, PA, PUR, PET	30 mm (original field samples ≥ 50 mm)	Fluorescence emission spectra provided distinguishable spectral patterns for seven polymer types. Excitation at 405 and 465 nm was most informative; 525 nm alone was insufficient for polymer differentiation. PP and PE could not be reliably distinguished from each other by spectral data alone. A low-cost imaging system (<USD 450) qualitatively validated the spectral patterns.	[122]
	340–380 nm, 450–490 nm, 515–560 nm	Solid particles dried in PTFE filters	PA, PE, PET, PP, PS, PUR, PVC	50–1200 μm	NR staining produced strong fluorescence in all seven polymers on PTFE filters. Plastics showed markedly higher fluorescence than nonplastic materials. PVC, PET and nylon fluoresced most intensely. PE and PP showed weaker but clearly positive signals. PP and PE were the most frequently confused polymer pair.	[125]
Nile Red and DAPI	430–490 nm (NR) 355–405 nm (DAPI)	Solid MP fragments and synthetic fibers River water and drinking water samples filtered on membrane filters	PP, HDPE, EPS, PVC (fragments) polyester, polyamide, acrylic (synthetic fibers)	NR	NR alone significantly overestimated MP abundance in river water and drinking water since H_2_O_2_ treatment did not effectively remove biological material that is also stained by NR. Co-staining with DAPI distinguished plastic particles from biological material, reducing false positives. Colored plastic fragments and dark-colored synthetics were not reliably stained by NR, highlighting a color-dependent limitation of the method.	[123]
LIF	405 nm	Laboratory water samples	PE, PP, PS, PET, PVC, PMMA, PUR, PC, Bakelite	<2 mm	LIF at 405 nm generated distinct fluorescence spectra for nine plastic types and seven nonplastic marine materials. Plastics exhibited a characteristic broad emission around 500 nm with an exponential spectral decay, distinguishable from natural organic materials that showed additional emission peaks near 680 nm. Raman emission peaks at 427 and 462 nm were also observed for plastics. Colored plastics, particularly red samples, produced additional spectral emissions that complicated identification.	[133]
	280 + 370 nm	Aerosols	PET, PP, PE, PS	1.2 μm (PETb) to 5.7 μm (PE)	MP emission maxima were located in the UV-A/UV-B range (285–365 nm), enabling discrimination from pollen grains, which emit at longer wavelengths. However, the three fluorescence channels of the WIBS were insufficient to distinguish between different polymer types. 3D excitation–emission maps suggested that instruments with additional UV channels and narrower emission bands could enable polymer-type discrimination.	[136]
FLIM	440 nm (FLIM, unstained) 405 nm (spectral characterization) 500 nm (NR-stained FLIM)	Laboratory samples	ABS, PET, PVC, PLA	100 μm	FLIM-phasor analysis generated distinct autofluorescence lifetime fingerprints for all four unstained MP types: ABS (2.0 ns), PET (2.2 ns), PVC (1.6 ns) and PLA (1.1 ns). Unstained autofluorescence lifetimes produced more distinct and stable phasor clusters than NR-stained measurements.	[112]

NR—not reported.

**Table 7:** Overview of FTIR and LDIR spectroscopy applications.

Matrix	Polymers Analyzed	Detection Limit (Min. Size)	Technique Used	Performance Metrics	Ref.
Wastewater	PE, PP, Nylon 6, PVC, PS	NR	FPA-μ-FTIR	An overall identification success rate of 98.33% on MP-spiked samples. A 47 mm filter can be fully mapped in under 9 h, compared to several days required by single-element detector FTIR.	[74]
Environmental samples	PVC, POM, PA, PC, PE, PET, PMMA, PP, PS, PU, PCL and PLA	NR	μ-FTIR	Showed 98% correct identification across a diverse dataset of environmental samples, including weathered and contaminated particles, without requiring prior cleaning.	[148]
Deep-sea sediments	PP-PE copolymer, PET and other polymers detected	100–4930 μm	μ-FTIR	The study found widespread MP contamination in deep-sea sediments and organisms of the Western Pacific, dominated by small fibrous particles mainly composed of PP-PE and PET.	[149]
Arctic sea ice and seawater beneath ice floes	PES, PA, PVC, and six additional polymer types in ice cores and PES, PA, PVC in surface waters	0.1–5 mm	FTIR	Most of the MPs found in the ice cores were PES and PA fibers, suggesting that an important source could be textiles or other fibrous products. The concentration of MPs ranged from two to 17 particles per liter of melted ice.	[150]
Lake sediments	PA, polyester (PES), PTFE, polyacetal (POM), PMMA, PP, PS, PVC, EVA, PLA	20–3384 μm	LDIR	Major polymers found were polyamide, polyester, PTFE, and polyacetal. Overall, 84% of MP particles had diameters below 100 μm.	[151]
Surface waters	PE, PP, PS, PVC, PET, PC, PLA, PAC, EVA, PMMA, PTFE, Nitrile rubber (RBR-N), Rubber (RBR)	20 μm	LDIR	The majority of MPs were <100 μm in diameter and classified as fragments. About half of all environmental samples were classified as fragments. Recovery of 88.3 ± 1.2%. Average analysis speed of 8 s per particle.	[152]

NR—not reported.

**Table 8:** Overview of representative HSI-based applications for MP analysis in complex matrices.

Matrix	Polymers Analyzed	Polymers Size	Performance Metrics	Ref.
Beach sand	PP, PE, PS, PVC, PET	>5 mm	Plastic detection from sand achieved sensitivity of 0.892–1.000 and specificity of 0.909–0.996, while polymer type classification reached sensitivity of 1.000 and specificity of 0.991–1.000. Multiple particles can be analyzed simultaneously, though performance in organic-rich or highly diverse matrices was not addressed in this study and may require further methodological refinement.	[157]
Beach sand	PE, PP, PA-6, PET, PS	<600 μm	Sensitivity and specificity exceeded 99% for all tested polymers, including weathered and colorless MPs below 600 μm that would otherwise be missed by visual inspection, without requiring prior sorting.	[108,109]
Fish gastrointestinal tract	PE, PP, PS, PC, PET	0.1–1 mm	Recall exceeded 98.8% and precision exceeded 96.2% for particles larger than 0.2 mm, with a total analysis time of approximately 6 min, eliminating the need for chemical digestion or manual separation.	[158]
Water	HDPE/LLDPE/MDPE/PP/PVC/UPVC/PET/PA/PS	NR	Identification was confirmed pixel-wise in a realistic water matrix simulant using a spectral feature table across ten polymer types, though no quantitative sensitivity or specificity values were reported. PA presented challenges in discrimination within mixtures due to its spectrally flat signature, and physically overlapping particles introduced additional confusion regions. The method is suitable for rapid on-site analysis without sample preparation.	[159]

NR—not reported.

**Table 9:** Overview of Raman spectroscopy applications for MP identification.

Matrix	Polymers Analyzed	Particle Size	Technique Used	Excitation Wavelength	Performance Metrics	Ref.
Seawater (microfluidic chip)	PS, PP, PE, PA, PET, PVC, PUR, PC, PMMA, CA (11 types)	PE (20–27 μm, 10–45 μm) PS (9.5–11.5 μm)	μ-Raman	532 nm and 785 nm	A CNN achieved 93% classification accuracy and a mean AUC of 98% for 11 polymer types from on-chip Raman spectra, outperforming SVM, RF, and ResNet34. Label-free identification of MPs below 50 μm was demonstrated directly in surface seawater.	[183]
Atmospheric aerosols	PS, PMMA	PS: 360 nm, 500 nm, 1, 2, 5 μm; PMMA: 360 nm, 500 nm, 2, 5 μm	SERS	785 nm	Single-particle detection of PS and PMMA was achieved down to 360 nm using SERS on a gold-sputtered substrate. Signal enhancement reached up to 2 orders of magnitude for PS analytes, enabling sub-micron identification without sample pretreatment.	[178]
Microfluidics (flow-through)	PS, PMMA, LDPE	Several tens to hundreds of μm	CARS + TPEAF	Pump: 650–900 nm	PS, PMMA and LDPE particles were selectively detected and classified in continuous flow at an average velocity of 4.17 mm/s. CARS provided chemical contrast for MPs while TPEAF simultaneously identified organic biotic particles, enabling real-time discrimination without filtration, separation, or labeling.	[182]
				Stokes: 1031 nm		
Commercial salts (17 brands, eight countries)	PE, PP, PA, PET, PS, PAN	>149 μm (mean: 515 ± 171 μm)	μ-Raman	785 nm	Of 72 particles extracted from 17 commercial salt brands across eight countries, 41.6% were confirmed as MPs by μ-Raman at 785 nm. PP and PE were the most frequently identified polymers. Mean particle size was 515 μm, with all confirmed particles above the 149 μm sieve threshold used during processing.	[184]
Bottled mineral water (particles on gold-coated filter)	PET, PES, PE, PA, PP	≥5 μm	μ-Raman	532 nm	μ-Raman at 532 nm identified MPs in three packaging types: reusable bottles (118 ± 88 particles·L^−1^), disposable bottles (14 ± 14 particles·L^−1^), and beverage cartons (11 ± 8 particles·L^−1^). Polymer type matched the packaging material in most cases, confirming packaging as the primary contamination source.	[185]
Dried particles (on glass)	PS, PET, PE, PVC, PP, PA6, PMMA, PC	100 nm–5 μm	μ-Raman (dual-PCA)	532 nm	Dual-PCA applied to Raman spectral matrices achieved near-perfect polymer assignment for eight polymer types across 100 nm–5 μm particle sizes, with correlations of 0.95–0.99. The method enabled fully automatic identification and 2D visualization of individual NPs, including weakly scattering particles where conventional library matching fails.	[186]
Dried particles	PS, PET, PE, PVC, PP, PA6, PMMA, PC, PUR, Teflon	NP (<1 μm) and microplastics down to 100 nm	μ-Raman (PCA + algebra + dual-PCA)	532 nm	A pipeline combining PCA, algebraic spectral merging, and dual-PCA enabled identification of MPs and NPs below 1 μm released from everyday plastic products. PCA eigenvalue score percentages provided semi-quantitative size estimation.	[187]
Dried samples (on AAO filters)	PE, PS, PP, PVC, PET	≥20 μm	μ-Raman (deep learning)	532 nm	A human–machine teaming approach achieved recall ≥ 99.4% and precision ≥ 97.1% for five polymers, reducing analysis time to <1 h per sample. Standalone deep learning at threshold 0.5 yielded recall > 98.4% but precision < 90%, insufficient for practical use.	[188]
Dried particles (on Al plate)	PE, PTFE, PS, PMMA, PVC	360 nm (smallest detected)	μ-Raman (RF)	532 nm	A random forest model trained on Raman spectra on highly reflective aluminum substrates achieved 98.8% average accuracy, 98.5% sensitivity and 100% specificity for particles down to 360 nm. Validation on spiked tap water exceeded 97% identification accuracy.	[189]
Dried particles (on glass slides)	PET, HDPE, PVC, LDPE, PP, PC, CPE	10–100 μm	μ-Raman (PCA + LDA, PCA + KNN, MLP)	785 nm	A multi-model ensemble combining PCA-LDA, PCA-KNN and MLP achieved > 98% classification accuracy. The reliefF feature selection algorithm was critical for distinguishing spectrally similar HDPE and LDPE.	[190]
Dry solid particles	ABS, PET, PBT, PS, POM, EVA, PMMA, PP, PC, LLDPE	10–500 μm	μ-Raman (CNN)	784–785 nm	A CNN model achieved 96.43% overall accuracy on standard samples and 95.6% on real environmental samples, outperforming SVM. The model demonstrated robustness to spectral variability introduced by additives, weathering and particle size heterogeneity.	[191]
Dried particles (on cover glass)	PS, PE, PP, PET, PMMA	1–10 μm	μ-Raman (CNN)	532 nm	A CNN classified MPs with 85% accuracy at only 0.4 s exposure per particle, approximately 67× faster than conventional full-mapping Raman spectroscopy. The approach directly addresses the low scattering cross-section bottleneck for sub-10 μm MP analysis.	[192]
Water solutions	PS, PET, PP, PE	33–161 nm	SERS (gold nanostars)	785 nm	Gold nanostar-based SERS substrates detected MPs in the sub-micron and nano regimes at concentrations as low as 625 ng·mL^−1^. LODs of 1.25 μg·mL^−1^ for 33 nm PS and 5 μg·mL^−1^ for 36 nm PET were achieved, improvements of ×16 for PS and ≥×4 for PET relative to previous AuNP sphere-based substrates. PP and PE showed reduced performance due to the absence of aromatic bonds.	[177]
Water solutions	PS	50–500 nm	SERS	785 nm	SERS with KI-aggregated Ag nanoparticles quantitatively analyzed PS nanoplastics, achieving an LOD of 6.25 μg·mL^−1^ for 100 nm PS with R^2^ ≥ 0.972 and analytical enhancement factors of 5.5 × 10^3^–2.3 × 10^4^. Recovery in spiked lake water ranged from 87.5 to 110% across different sizes and concentrations, confirming applicability to real environmental samples.	[193]

**Table 10:** Overview of representative algorithms for MP identification.

Algorithm	Identification Method	Dataset Size	Particle Size Range	Target Plastics	Performance Metrics	Ref.
Dual-PCA	μ-Raman	PE + PVC (7744 spectra) PA6 (900 spectra)	Sub-μm	PE, PVC, PA6	Dual-PCA automatically identified PE and PVC in a mixed laboratory sample and PA6 nanoplastics from trimmer debris, using PCA correlation values against reference spectra of eight common plastics. Preprocessing was required prior to analysis. Only PE, PVC, and PA6 were experimentally validated.	[186]
PCA + algebra-based merging + dual-PCA	μ-Raman	NR	NP (<1 μm)	PS, PET, PE, PVC, PP, PA6, PMMA, PC, PUR, Teflon	The pipeline merged multiple PCA images for cross-validation and improved SNR, enabling high-certainty identification of 10 polymer types. PCA eigenvalue score percentages provided estimates of relative MP/NP content within the scanned area.	[187]
Deep learning (single model + one-model-per-class)	μ-Raman	>64,000 spectra	≥20 μm	PE, PS, PP, PVC, PET	Standalone deep learning at threshold 0.5 achieved recall > 98.4% but precision < 90% for all classes (<80% for PET and PP), insufficient for practical use. Human–machine teaming (DL at threshold 0.1 followed by expert validation) achieved recall ≥ 99.4% and precision ≥ 97.1% for all five polymers, reducing analysis time from several hours to <1 h per sample.	[188]
PCA + RF (random forest)	LIF	2250 spectra	500 μm–1 mm (real samples) D50 < 8 μm	PE, PP, PS	The PCA-RF combined method achieved 99.7% composition identification accuracy for marine microplastic samples, with a correlation coefficient between predicted and actual mass concentrations exceeding 0.99. The approach resolved severely overlapping fluorescence spectra between different polymer types, enabling simultaneous identification of composition and quantification of mass concentration.	[134]
RDF	μ-FTIR (transmission)	3270 spectra	>10 μm	PE, PP, PMMA, PAN, PS	RDF classifiers achieved true positive rates of 94–100% for PE, PP, PMMA, PAN, and PS. Classification of one million spectra (1000 × 1000 pixel image, MPs ≥ 10 μm) was completed within minutes on a standard desktop PC.	[196]
KNN, SVM, decision trees	EIS	NR	500–4000 μm	PET	K-NN was the best performer among three classifiers tested, achieving 86.6% accuracy for PET MPs in pure water conditions. When applied to complex water matrices simulating real environmental conditions (organic and inorganic interferents), K-NN accuracy improved to 89.2%, compared with 81.2% for SVM and 86.0% for decision trees.	[79]
RF (random forest)	μ-Raman	1200 spectra (200 per class + 200 blank)	360 nm (smallest detected for PS and PMMA)	PE, PTFE, PS, PMMA, PVC	A random forest model trained on Raman spectra of PE, PTFE, PS, PMMA and PVC nanoplastics on highly reflective aluminum substrates achieved 98.8% average accuracy, 98.5% average sensitivity and 100% average specificity for particles down to 360 nm. Validation on spiked tap water exceeded 97% identification accuracy. Real rainwater samples yielded successful detection of nanoscale PS and PVC.	[189]
PCA + LDA, PCA + KNN, MLP (six layers)	μ-Raman	4900 spectra	10–100 μm	PET, HDPE, PVC, LDPE, PP, PS, CPE	A multi-model ensemble combining PCA-LDA, PCA-KNN and MLP achieved > 98% overall classification accuracy for seven household polymer types across standard, real and environmentally stressed samples (up to 99.3% for standard samples). The reliefF feature selection algorithm was critical for distinguishing spectrally similar HDPE and LDPE.	[190]
CNN	μ-Raman	1400 spectra (reference) + 1080 (treated) + 180 (environmental)	NR	ABS, PET, PBT, PS, POM, EVA, PMMA, PP, PC, LLDPE	A CNN model trained on 1660 Raman spectra of 10 polymer types achieved 96.43% accuracy on standard samples and 95.6% on real environmental samples, outperforming SVM. The model demonstrated robustness to spectral variability introduced by additives, weathering and particle size heterogeneity across the 10–500 μm range.	[191]
CNN	μ-Raman	72,000 spectra	1–10 μm	PS, PE, PP, PET, PMMA	A CNN with tailored spectral interpolation classified MPs in the 1–10 μm range with >80% accuracy at 0.4 s exposure per particle, approximately 67× faster than conventional full-mapping Raman spectroscopy.	[192]
CNN	FTIR	NR	NR	PE, PP, PS	A 1D-CNN trained on FTIR spectra of virgin and UV-aged PE, PP and PS achieved 100% identification accuracy for aged commercial MP samples, compared with 80% for a deep neural network, 60% for random forest and 40% for an artificial neural network. The CNN greatly simplified preprocessing, preserving raw spectral details without additional noise or distortions, and demonstrated superior robustness to changing or fuzzy feature peaks introduced by UV aging.	[197]
PDM + PIM	Nile Red	96 PDM + 168 PIM	50–1200 μm	PA, PE, PET, PP, PS, PUR, PVC	Two supervised machine learning models trained on RGB fluorescence images of Nile Red-stained MPs achieved 95.8% accuracy in distinguishing plastic from nonplastic particles (PDM) and 88.1% accuracy in identifying polymer type (PIM) for virgin reference samples. When applied to spiked environmental matrices (seawater and biota), detection accuracy was 92.7% and polymer identification accuracy was 80%.	[125]

NR—not reported.

## Data Availability

No new data were created or analyzed in this study. Data sharing is not applicable to this article.

## Abbreviations

The following abbreviations are used in this manuscript:

AAO	Anodic aluminum oxide
ABS	Acrylonitrile butadiene styrene
ACF	Acoustic contrast factor
ANN	Artificial neural network
ATR	Attenuated total reflectance
ATR-FTIR	Attenuated total reflectance–Fourier transform infrared spectroscopy
BAW	Bulk acoustic wave
CARS	Coherent anti-Stokes Raman scattering
CCD	Charge-coupled device
CMOS	Complementary metal–oxide semiconductor
CNN	Convolutional neural network
CPE	Chlorinated polyethylene
DEP	Dielectrophoresis
DUV	Deep ultraviolet
EIS	Electrical impedance spectroscopy
EVA	Ethylene-vinyl acetate
FFF	Field-flow fractionation
FLIM	Fluorescence lifetime imaging microscopy
FPA	Focal plane array
FTIR	Fourier transform infrared spectroscopy
HDPE	High-density polyethylene
HSI	Hyperspectral imaging
HSI-NIR	Near-infrared hyperspectral imaging
IR	Infrared
KNN	K-nearest neighbors
LATR-FTIR	Large-area attenuated total reflectance–Fourier transform infrared spectroscopy
LDA	Linear discriminant analysis
LDIR	Laser direct infrared spectroscopy
LDPE	Low-density polyethylene
LIF	Laser-induced fluorescence
LLDPE	Linear low-density polyethylene
LOC	Lab-on-a-Chip
LOD	Limit of detection
LSPR	Localized surface plasmon resonance
MLP	Multilayer perceptron
MP	Microplastic
NIR	Near-infrared
NP	Nanoplastic
NR	Not reported
OPTIR	Optical photothermal infrared spectroscopy
PA	Polyamide
PA6	Polyamide 6 (Nylon 6)
PAC	Polyacrylate
PAN	Polyacrylonitrile
PBT	Polybutylene terephthalate
PC	Polycarbonate
PCA	Principal component analysis
PCL	Polycaprolactone
PDM	Plastic detection model
PDMS	Polydimethylsiloxane
PE	Polyethylene
PES	Polyester
PET	Polyethylene terephthalate
PIM	Polymer identification model
PLA	Polylactic acid
PMMA	Poly(methyl methacrylate)
POM	Polyoxymethylene (Polyacetal)
PP	Polypropylene
PS	Polystyrene
PTFE	Polytetrafluoroethylene
PUR	Polyurethane
PVC	Polyvinyl chloride
RBR	Rubber
RBR-N	Nitrile rubber
RDF	Random decision forest
ResNet	Residual neural network
RF	Random forest
RPS	Resistive pulse sensing
RRS	Resonance Raman spectroscopy
SERDS	Shifted excitation Raman difference spectroscopy
SERS	Surface-enhanced Raman scattering
SRS	Stimulated Raman scattering
SVM	Support vector machine
TERS	Tip-enhanced Raman spectroscopy
TPEAF	Two-photon excited autofluorescence
μ-FTIR	Micro-Fourier transform infrared spectroscopy
μ-Raman	Micro-Raman spectroscopy
UV	Ultraviolet
